# Mesoscopic Structures and Coexisting Phases in Silica Films

**DOI:** 10.1021/acs.jpcc.1c10216

**Published:** 2022-02-11

**Authors:** Kristen M. Burson, Hyun Jin Yang, Daniel S. Wall, Thomas Marsh, Zechao Yang, David Kuhness, Matthias Brinker, Leonard Gura, Markus Heyde, Wolf-Dieter Schneider, Hans-Joachim Freund

**Affiliations:** † Hamilton College, 198 College Hill Road, Clinton, New York 13323, United States; ‡ Fritz-Haber-Institut der Max-Planck-Gesellschaft, Faradayweg 4-6, 14195 Berlin, Germany

## Abstract

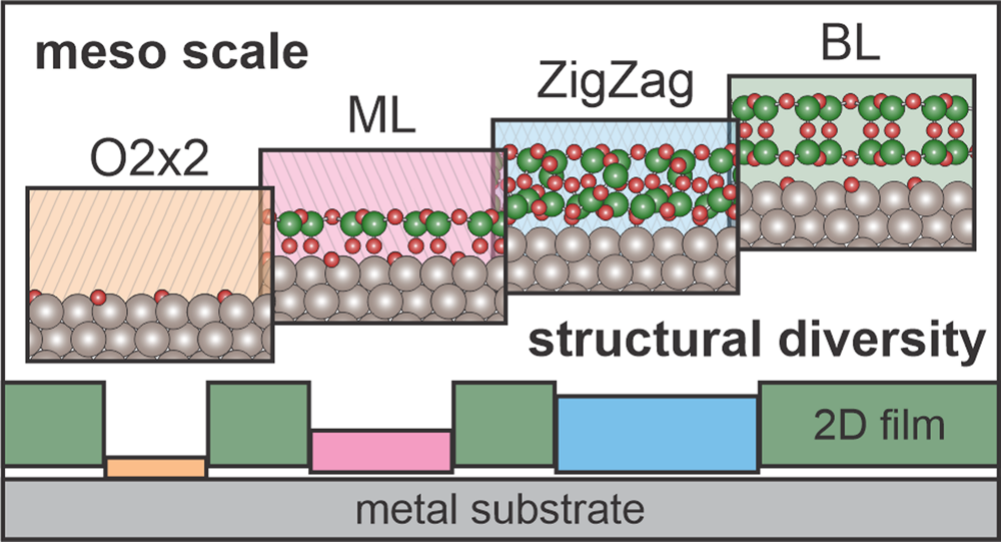

Silica films represent a unique two-dimensional film system, exhibiting both crystalline and vitreous forms. While much scientific work has focused on the atomic-scale features of this film system, mesoscale structures can play an important role for understanding confined space reactions and other applications of silica films. Here, we report on mesoscale structures in silica films grown under ultrahigh vacuum and examined with scanning tunneling microscopy (STM). Silica films can exhibit coexisting phases of monolayer, zigzag, and bilayer structures. Both holes in the film structure and atomic-scale substrate steps are observed to influence these coexisting phases. In particular, film regions bordering holes in silica bilayer films exhibit vitreous character, even in regions where the majority film structure is crystalline. At high coverages mixed zigzag and bilayer phases are observed at step edges, while at lower coverages silica phases with lower silicon densities are observed more prevalently near step edges. The STM images reveal that silica films exhibit rich structural diversity at the mesoscale.

## Introduction

As is well-known in zeolite catalysis,^1,2^ performing reactions in confined spaces presents an exciting route to influencing reaction rates and selectivity. Substrate-supported two-dimensional (2D) materials have recently garnered the interest of the scientific community as ideal model systems for studying confined space reactions due to their well-defined spatial confinement parameters and relative structural simplicity which enable comparison with theoretical models.^3−12^ Theoretical and experimental studies have explored the impact of such spatial confinement on reaction rates, activation energies, and adsorption energies for common molecular reactions such as CO oxidation, water formation, and the hydrogen evolution reaction. The activation energy of CO oxidation is reduced for both graphene/Pt(111)^3^ and BN/Pt(111)^4^ compared to bare Pt(111). Water formation from H_2_ and adsorbed oxygen on Ru(0001) exhibits a lower apparent activation energy^5^ and an accelerated rate^6^ when confined beneath a two-dimensional vitreous silica bilayer, BL-silica/Ru(0001), as compared with the same reaction on bare Ru(0001). Because of the coupled kinetics of adsorption, reaction, and diffusion, the explanation of those observations is not straightforward and requires detailed kinetic studies.^13^ However, the study of the silica film system presents an opportunity to do so under well-defined conditions applying surface-sensitive techniques. In addition, because the reaction rates can be influenced by the degree of confinement, rates of intercalation, and the presence of structural defects in thin films,^14−18^ interpreting the results of confined space reaction studies necessitates a detailed understanding of the confinement structures themselves at multiple length scales.

Oxide systems are known to exhibit structural diversity, with structures that can be very sensitive to the particular preparation conditions. Quasi-crystalline phases of 2D barium titanate develop through high-temperature annealing of BaTiO_3_(111) thin films on Pt(111); two-dimensional layers of the ternary oxide iron tungstenate grown on Pt(111) present unconventional (2 × 2) and (6 × 6) honeycomb structures distinct from bulk structure elements. For La_0.8_Sr_0.2_MnO_3_ (110) complex oxides, small composition changes can result in a rich range of surface reconstructions.^19,20^ In these material systems, unique structural phases can coexist depending on thickness variation, annealing temperature, or composition.^21^ Similarly, silica bilayer films can exhibit multiple structural phases, and numerous detailed studies have thoroughly characterized the material structure on the nanometer and atomic scales.^22−28^ Some structural analyses, such as those exploring ring size distributions and ring neighborhoods, rely on data at the nanometer scale, while other analyses, such as spectroscopic studies of bonding structures and crystal structure from diffraction, draw from data sets collected by using spatially averaged techniques. However, detailed studies at the mesoscale are still lacking. Given the influence of material structure on confined space reactions, it can be expected that pristine films of a single phase will behave differently from films that exhibit structural diversity.

In this paper, we present a characterization of structural diversity at the mesoscale in silica films grown on Ru(0001) using low-temperature scanning tunneling microscopy (LT-STM). We discuss the known phases of silica and demonstrate that various phases of silica may coexist. We look at the role of step edges in influencing film structure. Finally, we consider the prevalence and influence of holes in silica structures as a function of sample coverage. These metrics provide a detailed picture of the structure of silica at the mesoscale, which provides additional information to enable rational understanding of future confined space reaction studies.

## Experimental Methods

Silica films were prepared on a clean Ru(0001) substrate in ultrahigh vacuum. Ru(0001) surfaces were cleaned with cycles of Ar^+^ sputtering and annealing in UHV at 1450 K. A 3O-(2 × 2)-precover on Ru(0001) was established by annealing at 1180 K in 10^–6^ mbar of O_2_ for 10 min. Silicon was deposited from a rod via an e-beam evaporator in 2 × 10^–7^ mbar of O_2_, and the sample was subsequently annealed to 1180 K in 2 × 10^–6^ mbar of O_2_ for 20 min. Finally, samples were cooled to below 500 K in an oxygen environment for 20–30 min. Eight distinct films were prepared with these procedures; specific modifications for individual samples, such as variations in the annealing conditions or cooling rate, are noted in the text. Following film preparation and subsequent verification of silica structures with LEED and Auger, STM images were taken with a scanning tunneling microscope operated at low temperature (∼5 K). Previous studies have extensively explored the sample preparation phase space;^29^ therefore, this current study does not pursue a systematic exploration of different preparations but rather seeks to characterize the structural aspects of coexisting phases from films with controlled preparation conditions.

## Results and Discussion

**Figure 1:**
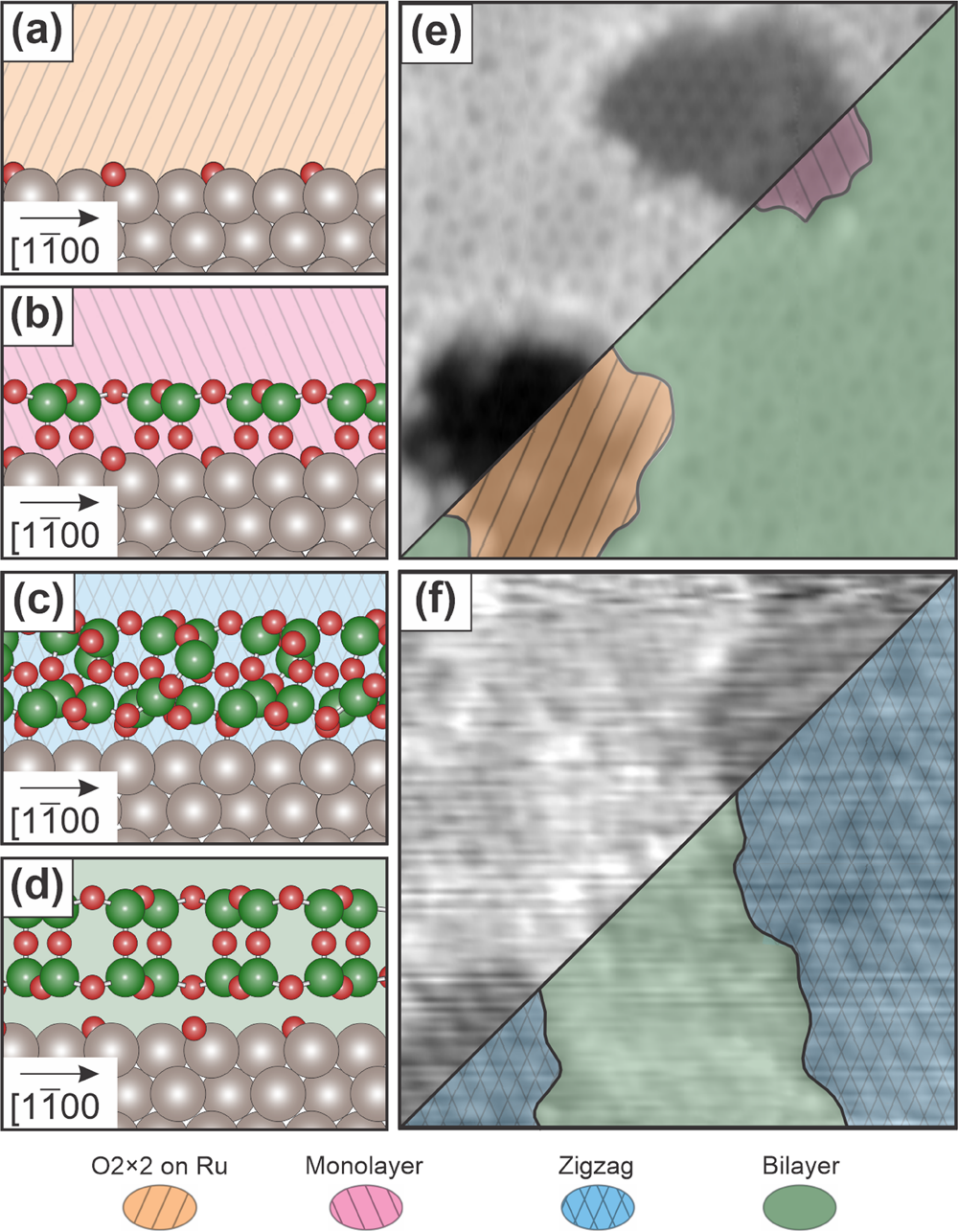
Coexisting silica structures grown on Ru(0001). Side views are shown with atomic models of possible silica structures (a–d), and top-view terraces are show with scanning tunneling microscopy images (e, f). Color-coded overlays on the lower right of the STM images and as the background color for the atomic models indicate the four commonly observed structures: (a) O(2 × 2) on Ru(0001), yellow stripes; (b) monolayer silica, pink stripes; (c) zigzag phase silica, blue diamonds; and (d) bilayer silica, solid green. STM images show the coexistence of multiple phases from films with sub-bilayer coverage: (e) Holes incorporated in bilayer silica, a hole with 3O(2 × 2)-Ru (left) and a hole with monolayer silica (right); scale 11.5 nm × 11.5 nm, *V*_S_ = 0.1 V, *I*_T_ = 10 pA. (f) Zigzag patches with bilayer silica: scale 11.5 nm × 11.5 nm, *V*_S_ = 1.0 V, *I*_T_ = 30 pA.

A diverse set of structures has been observed to date in thin silica films on Ru(0001) substrates, and the atomic-scale features of these polymorphs have been well characterized. Atomic models summarizing known silica polymorphs on Ru(0001) from previous literature are shown in Figure 1: monolayer silica films (ML), bilayer silica films (BL), and zigzag phase silica films (ZZ). These films were each grown on an oxygen-precovered Ru(0001) substrate with a 3O (2 × 2) structure (Figure 1a) with sub-bilayer silica coverages. Each silica structure consists of a network of interconnected SiO_4_ tetrahedral building units. Bonding between these units and the Ru(0001) substrate for ML and ZZ films leads to different Si:O stoichiometries: SiO_2.5_ for ML films, SiO_2.17_ for ZZ films, and SiO_2.0_ for the silica bilayer. For ML silica films (Figure 1b), a single oxygen atom from each tetrahedral unit is chemically bound directly to the Ru(0001) substrate and bridges to the silicon atom. The lattice constant for the silica monolayer structure is commensurate with the Ru(0001) lattice.^25^

A second phase of silica, BL silica (Figure 1d), is produced with two monolayers of coverage (2 MLs) and consists of a fully saturated bonding structure in which a bridging oxygen atom connects the top and bottom layers of the bilayer structure.^22,23^ This structure is chemically decoupled from the substrate, attracted only by van der Waals forces; the weak bonding with the substrate provides for facile transfer of the resulting bilayer films between substrates through a lift-off process. Bilayer silica films can exhibit either crystalline or vitreous structures and additionally show a diverse complement of domain boundaries and point defects.^25,30^

Finally, a novel zigzag phase has been observed (Figure 1c), which exhibits intermediate substrate coupling with material coverage (∼2 MLs) comparable to bilayer silica. The zigzag phase is metastable and can be transformed to the silica bilayer structure upon annealing at higher temperatures.^24^Figures 1e and 1f show that these diverse phases of silica can coexist at the mesoscale. In Figure 1e, a silica bilayer film with coverage less than two full monolayers, prepared following the procedure outlined in the Experimental Methods section, contains several holes. One hole connects to a region with a monolayer film (top right), and another exposes the oxygen-covered Ru(0001) substrate (bottom left). A domain boundary can be seen in the crystalline monolayer region. In Figure 1f, we observed a closed film (e.g., complete 2 MLs coverage), composed of a mix of vitreous bilayer silica and the metastable zigzag phase. The coexistence of these various phases of silica at the mesoscale may be a key for different chemical reaction pathways. The interface between the structures might be of importance due to differently favored reactions. Additionally, holes and vitreous structure could enable intercalation and diffusion for confined space reactions,^14,17,18^ which will depend on the ring sizes present and on the size and shape of holes. Step edges also play a role in influencing film structure and growth. Here, we first explore the role of step edges and then turn our attention to holes.

In a previous study, the continuous coverage across single step edges for both vitreous and crystalline bilayer films was emphasized.^31^ Klemm et al. explored mesoscale structures in the presence of large step bunches and concluded that monolayer phases were prevalent near these step bunches.^32^ Because those studies were performed with LEEM/PEEM, it was not possible to discern individual atomic steps or the prevalence of coexisting phases with smaller domain sizes. Here we extend the previous observations by looking at mesoscale structural diversity in the presence of atomic scale single steps and using STM to examine smaller domain sizes.

**Figure 2:**
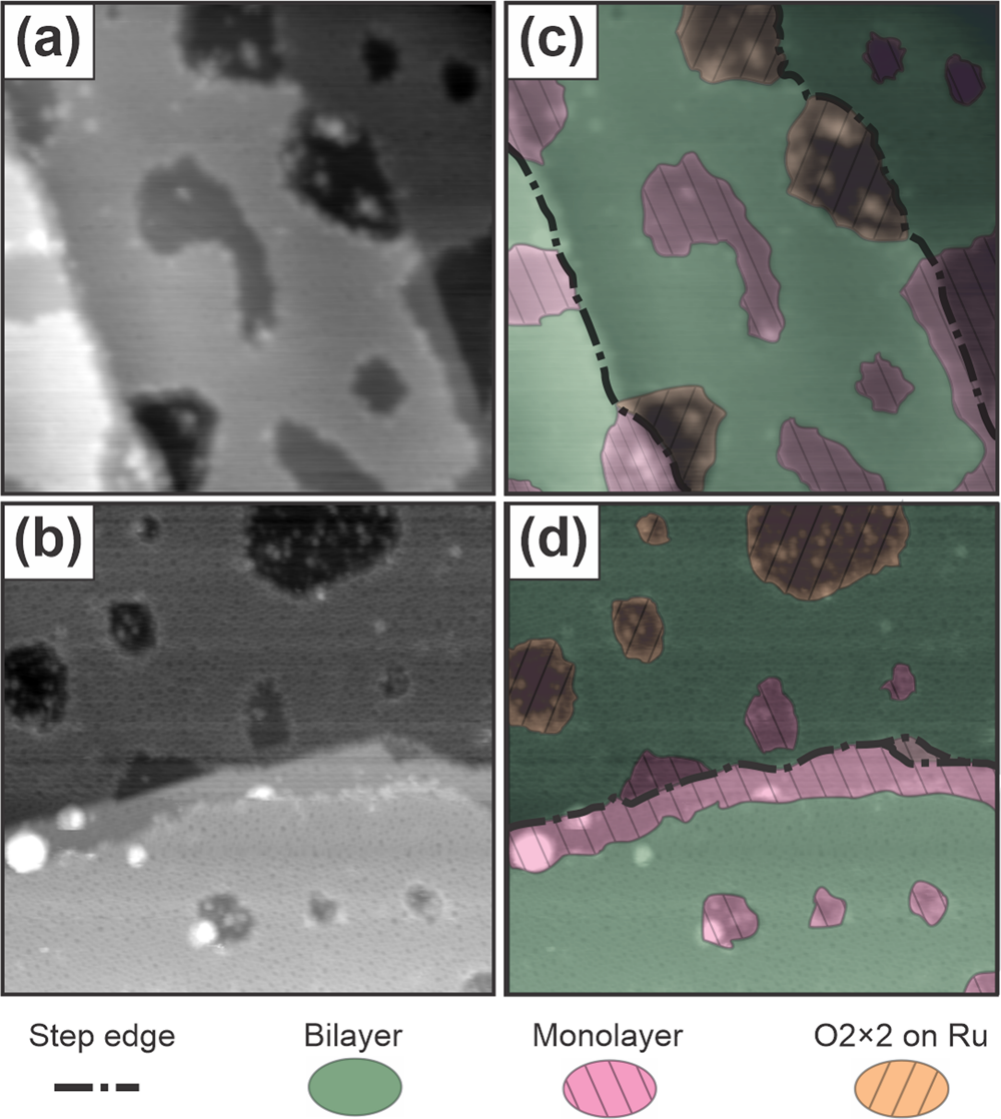
Overview STM images of observed silica structures with step edges (a, b) and color-coded images (c, d). Scale: 40 nm × 40 nm. (a, c) *V*_S_ = 1.5 V, *I*_T_ = 20 pA; (b, d) *V*_S_ = 2.0 V, *I*_T_ = 50 pA.

Figure 2 shows coexisting silica film phases grown on Ru(0001) with substrate steps indicated with a black dashed line. In general for two-dimensional materials, step edges may act as nucleation sites for film growth, with film growth often nucleating at the bottom of the step edge, which provides multiple anchoring sites for the atom. For example, graphene grown on transition metals nucleates at step edges,^33,34^ and graphene grown epitaxially on highly stepped silicon carbide can exhibit pinning at step edges.^35^ Two-dimensional films grown on substrates can alternatively exhibit carpet growth, flowing across the step edge. Therein, the atomic scale steps in the substrate have little to no impact on the atomic scale structure of the two-dimensional film; this is the case for carpet growth of graphene.^36^ DFT results from a recent detailed analysis for closed silica bilayer films found that the stoichiometry at the step may exhibit smaller Si:O concentrations due to pinning to substrate step edges.^31^

For all of the images shown in Figure 2, the step influences the film growth, evidenced by the presence of noncontinuous phases across the step edges. In Figures 2a,c and 2b,d, a combination of bilayer, monolayer, and oxygen-covered Ru regions can be observed. Some monolayer holes and substrate holes are observed within silica bilayer regions on the Ru(0001) terraces while other holes terminate at the step edge. On the upper terraces of the step edges in particular, monolayer and substrate holes are especially present. In Figure 2b,d, a monolayer silica film region covers the entire step length of the upper terraces near the step edges. This is consistent with the observations of reduced silicon concentrations near step bunches after annealing^32^ and indicates that even a single step can influence the mesoscale structure of bilayer silica films.

Even as we conclude that the presence of the step influences the film structure, it should also be noted that silica coverage has been found to be continuous across the step.^31^ Several additional studies point to the continuity of the silica films. First, the structure of bilayer silica films is retained after film transfer to a new substrate by PMMA mechanical exfoliation,^25^ indicating film stability. Second, the water formation reaction front between silica and a Ru(0001) substrate evolves continuously across step edges.^5^ This indicates that water formation, i.e., chemical bonding, is not hindered by the presence of these step edges or by interactions between the step edge and the silica film.

**Figure 3:**
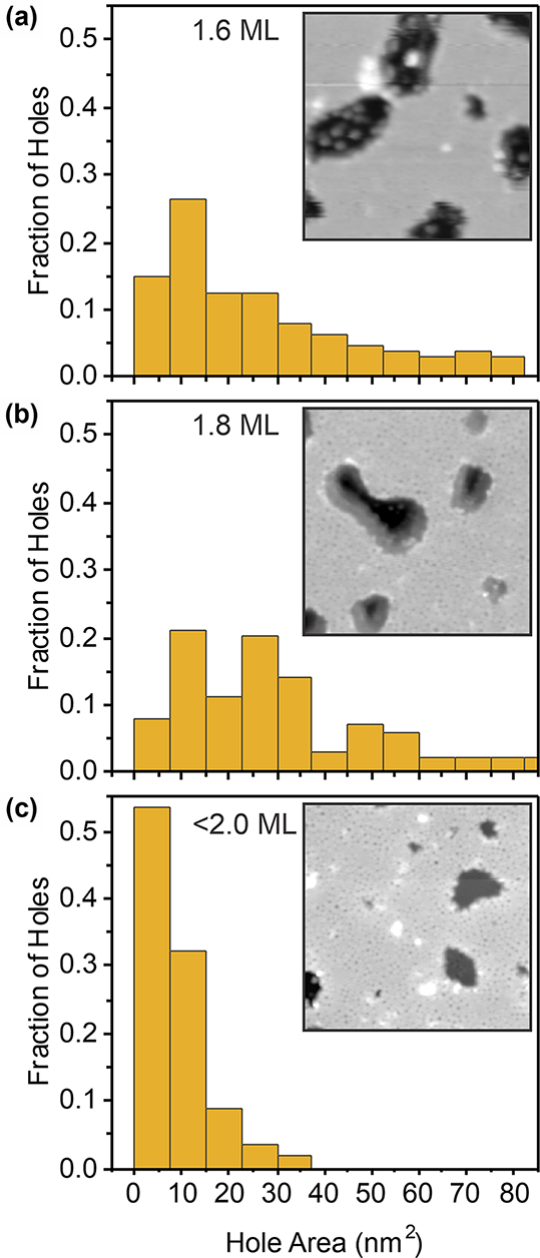
Histograms showing the distributions of hole areas for three different silica film coverages. Silica films represent three distinct sample preparations, all prepared by using the standard procedure described in the Experimental Methods, with variations in the amount of silicon deposited. In each case, the bin height represents the fraction of the total number of holes which exhibit the binned range of areas. STM images at each coverage are shown as insets. Scale 15 nm × 15 nm; (a) *V*_S_ = 2.0 V, *I*_T_ = 10 pA; (b) *V*_S_ = 3.0 V, *I*_T_ = 10 pA; (c) *V*_S_ = 3.0 V, *I*_T_ = 10 pA.

Step edges are one mesoscale feature that can influence coexisting phases in silica film structures; holes are another. Previous studies report holes in silica films from 10 to 100 nm in diameter;^25,32^ here we assess how the hole size varies as a function of coverage. Figure 3 shows histogram distributions of hole areas within silica bilayers as a function of sample coverage. Each sample was prepared by using the standard recipe, varying only the amount of silicon deposited. All samples considered in the analysis of holes consist of >80% bilayer coverage across the sample. Monolayer patches may be present in holes. At lower sample coverages, the holes exhibit a wide distribution of areas. With increasing coverage, fewer large area holes are present, and higher coverages, near 2.0 ML, include only small area holes.

Holes in bilayer silica can expose either the underlying, O-covered Ru(0001) or monolayer patches, and this too depends on coverage. Most holes for the sample with 1.6 ML coverage expose the 3O-(2 × 2)-Ru substrate. By contrast, most holes in the <2.0 ML coverage sample connect to monolayer patches, as shown in the inset STM image in Figure 2 (bottom). At this coverage, only a single hole exposing the substrate is observed, intersecting the leftmost image border. At an intermediate coverage, both monolayer patches and substrate holes are observed, as can be seen in the 1.8 ML sample and in Figure 1e.

Hole shapes range from round to oblong and typically exhibit smooth perimeters. These holes have been observed to remain even when films are transferred out of vacuum and imaged with AFM in liquid environments, indicating that both the holes and the film structure are quite stable.^25^ Larger holes in silica films have been observed with LEEM. Therein, dendritically shaped and elongated holes in the crystalline silica bilayers with a width on the order a 100 nm have been reported.^32^ In addition to characterizing the size and shape of holes, we considered the relationship between holes and the atomic scale features of the surrounding silica structures.

**Figure 4:**
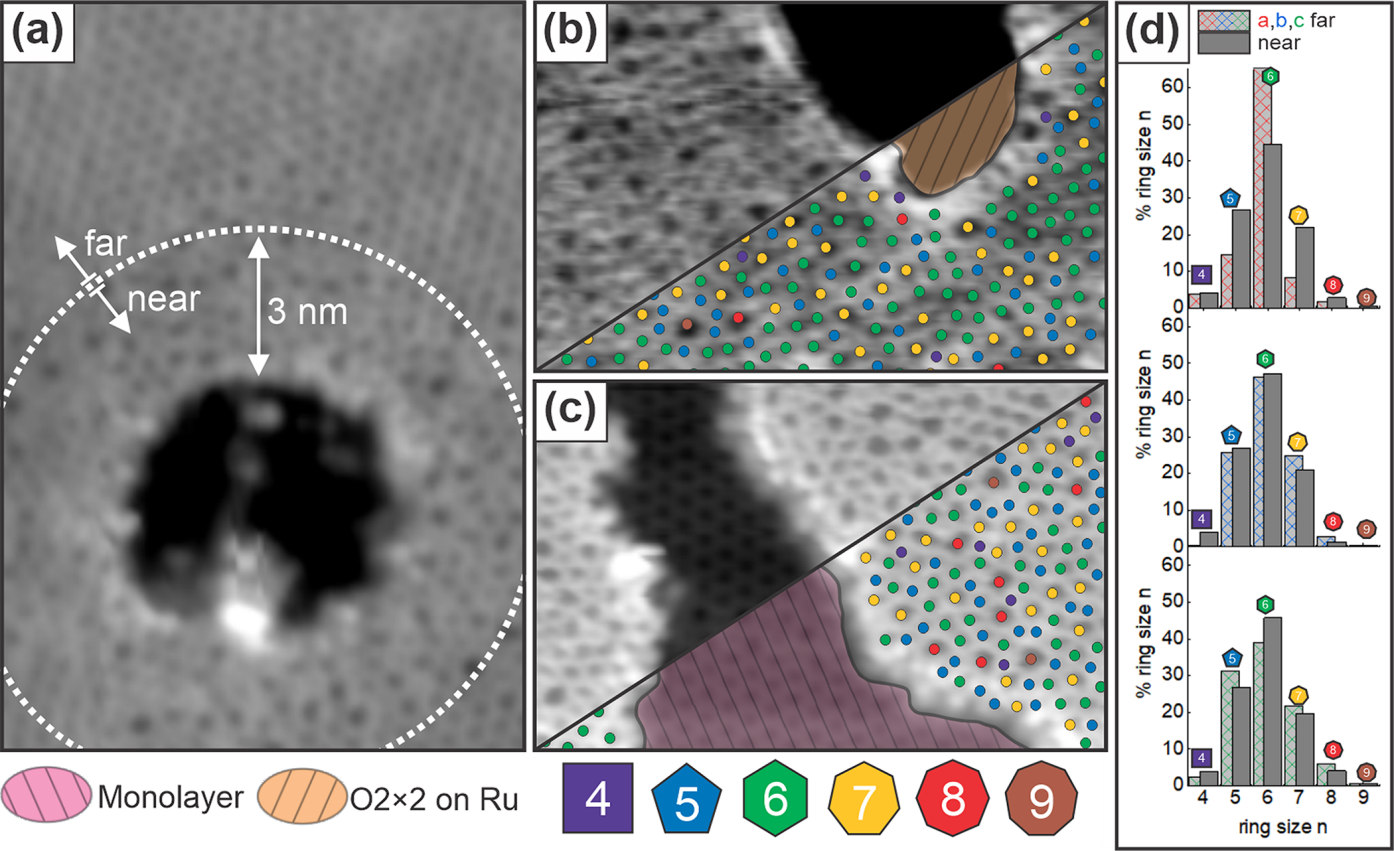
STM images showing ring-size distributions around (a) a hole exposing O(2 × 2)-Ru located within a predominantly crystalline region of a silica bilayer film, (b) a hole exposing O(2 × 2)-Ru located within a vitreous bilayer silica region, and (c) a hole filled with ML silica. Ring sizes, defined by the number of neighboring rings, are indicated as an overlay with colored circles in (b) and (c). (d) Statistical distribution of ring sizes near and far from the holes in images (a–c). For all images, the region far from the hole exhibits comparable or greater crystallinity than the region near the hole. Scale (a) 10.4 nm × 17.9 nm, *V*_S_ = 2.0 V, *I*_T_ = 70 pA; (b) 11.4 nm × 7.5 nm, *V*_S_ = −1.0 V, *I*_T_ = 10 pA; (c) 11.4 nm × 7.5 nm, *V*_S_ = 1.0 V, *I*_T_ = 10 pA.

Figure 4 displays a ring size analysis for three different types of holes: a hole that exposes the substrate within a largely crystalline region of a bilayer film (Figure 4a), a hole that exposes the substrate within a vitreous region of bilayer silica (Figure 4b), and a hole that connects to the monolayer within a vitreous region of bilayer silica (Figure 4c). The data come from three distinct samples prepared by using the standard procedure described in the Experimental Methods section, varying only the annealing time and temperature for the oxidation step. Sample 4a was annealed for 15 min at 1230 K, sample 4b^33^ for 20 min at 1130 K, and sample 4c for 50 min at 1120 K. Ring size distributions are determined by using a semiautomated network detection scheme (see ref (31) and the Supporting Information). The distributions are considered close to the hole, defined to include rings within 3 nm of the hole perimeter, and far from the hole, rings beyond 3 nm, in order assess changes in the atomic scale structure of silica bilayers near the hole perimeter. The coordinates of component atoms for each ring are determined to account for partial ring counts near the boundaries of each region. These coordinates are provided in the Supporting Information. In each case, the same numbers of atoms are included to establish ring statistics near the hole and far from the hole, and the crystallinity is calculated. The crystallinity is defined as the fraction of six-membered rings with respect to the total number of rings. The results of this analysis are summarized in Table 1. Amorphous network structures were further confirmed by using a log-normal distribution analysis (see the Supporting Information).^37^ In all cases, a comparable or lower degree of crystallinity, e.g., more vitreous structure, is observed near the hole perimeter.

**Table 1:** Comparing the Crystallinity Values far and near from Hole Structures in Silica Films^a^

	crystallinity far from hole (*C*_*n*_)	crystallinity near hole (*C*_*n*_)	no. of Si atoms
4a	0.71 ± 0.04	0.44 ± 0.04	605
4b	0.46 ± 0.04	0.47 ± 0.04	310
4c	0.38 ± 0.04	0.45 ± 0.04	654

a The numbers of Si atoms included in the analysis are provided to establish the extent of statistical representation.

For all three examples, the crystallinity near the hole perimeter reflects typically observed values for vitreous silica films (*C* = 0.42);^38^ an additional ten images with holes have been identified in both vitreous and crystalline silica regions, each showing vitreous behavior around the hole. One possible explanation is that the loose boundary condition may template vitreous structure. A particularly interesting case is that of the monolayer hole in Figure 4c. Here the monolayer exhibits a crystalline structure as a result of direct bonding to the commensurate Ru(0001) substrate and yet is surrounded by the vitreous bilayer.

Depending on the film growth mechanism, one might hypothesize that monolayer crystalline structure would template the growth of surrounding bilayer structures that are also crystalline. However, the fact that this is not observed provides some insight into the film growth mechanisms. In previous work it has been reported that holes form during the annealing process and that, once formed, these holes cannot fill up through additional silicon deposition and subsequent oxidation.^32^ Nonetheless, the change in crystallinity for the bilayer near the hole edge compared to “inland” bilayer ring distributions suggests that structural rearrangements of the silica bilayer near hole boundaries can occur during the hole formation process or may indicate that holes preferentially form in regions with lower local crystallinity. A previous study by Malashevich et al. using density functional theory concluded that deviations from the crystalline hexagonal structure would be expected at lower SiO_2_ density in bilayer silica due to surface and interfacial tensions.^39^ This conclusion is consistent with the experimental observation here showing the transition from a crystalline region to an amorphous region near the hole.

## Conclusion

By looking at films at the mesoscale, it is evident that multiple structures can coexist within a single film including monolayer, bilayer, and zigzag phases, crystalline and vitreous structures, and holes of varying depths and sizes. Characterizing this structural diversity at the mesoscale can provide key insights for interpreting experiments on confined space chemistry between silica films and their substrates, where structure–reactivity relationships play an important role. On the theoretical side, the formation of mesoscale holes and the amorphization of the structure near the holes are beyond the present possibilities of atomistic simulations at the level of density functional theory calculations. In principle, plots of thermodynamic stability of various silica phases as a function of an external parameter, for example, the oxygen pressure, could shed some light on the origin of these mesoscopic structures. But in this case a large number of possible structures of O atoms at the interface have to be considered, with the obvious conclusion that at high partial pressures of oxygen phases with more O at the interface are preferred, and vice versa.

Here we have shown experimentally that the presence of holes and step edges can influence the atomic-scale film structure, which in turn impacts the intercalation and diffusion pathways for reactive molecules. An attentiveness to mesoscale structural diversity is necessary for rational understanding of reactivity studies. Additional explorations are needed to establish the influence of interfacial oxygen on mesoscale structure as this can decouple the silica film from the growth substrate and change the spatial confinement between film and substrate. Different growth substrates can also influence mesoscale structure through varied oxygen affinity and compressive strain,^40,41^ and studies of mesoscale structure for other substrate supported two-dimensional films, such as germania,^42^ may reveal ideal combinations for confined space reaction studies. The influence of step edges on atomic scale film structure also presents an exciting route for templating desired mesoscale structures by using tailored vicinal surfaces.

## Supporting Information Available

Log-normal probability plots for ring distributions near and far from the holes shown in Figure 3; semiautomated network detection description; table of Si atom positions (PDF)
