# Supplementary material for: Mesoscopic Structures and Coexisting Phases in Silica Films
Source: J Phys Chem C Nanomater Interfaces. 2022 Feb 11;126(7):3736–42. doi: 10.1021/acs.jpcc.1c10216 (PMC8883523; doi:10.1021/acs.jpcc.1c10216)
Supplement: Supplementary file 1 — jp1c10216_si_001.pdf [file jp1c10216_si_001.pdf]

Supplemental Information for

Mesoscopic Structures and Co-existing Phases in Silica Films

Kristen M. Burson,[1] Hyun Jin Yang,[2] Daniel S. Wall,[1] Thomas Marsh,[1] Zechao Yang,[2] David Kuhness,[2] Leonard Gura,[2] Markus Heyde,[2] Wolf-Dieter Schneider,[2] and Hans-Joachim Freund[2*]

1) Hamilton College, 198 College Hill Road, Clinton, NY, 13323 USA
2) Fritz-Haber-Institut der Max-Planck-Gesellschaft, Faradayweg 4-6, 14195 Berlin, Germany
* Email: freund@fhi.mpg.de

Index

1. Log-normal Distribution Plots 1
2. Semi-automated Network Detection 2
3. Si Atom Positions 3

1. Log-normal Distribution Plots

Figure S1 shows the log-normal probability plots for ring distributions near and far from the holes shown in Figure 3 of the main manuscript. Amorphous bilayer silica have been shown to follow a log-normal distribution. The log-normal distribution was predicted by Zachariasen's random network theory for two-dimensional, tetrahedrally coordinated systems and has been verified experimentally for the amorphous silica bilayer. Each set of ring statistics, both near and far from the holes exhibit a log-normal distribution, which appears linear when plotted on a log-normal probability plot. The one exception is the highly crystalline region far from the hole in Figure 1a (red, ×).

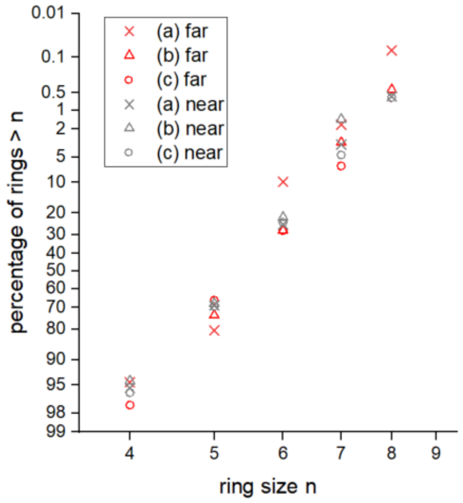

Figure S1: Lognormal probability plot of the ring sizes from Figure 3 data. Grey markers correspond to distributions in regions near the holes while red markers correspond to regions far from the hole. With the exception of the highly crystalline region (a, far), each distribution exhibits linear behavior on the log-normal probability plot.

2. Semi-automated Network Detection

Figure S2 illustrates the pathway of our semi-automated network detection on a small network area of 4 x 4 nm² from Figure 3c in the main manuscript. First, the ring center positions are identified. The center positions are shown in Figure S2a. Secondly, the neighborhood of each ring center is determined with an image segmentation algorithm that takes the contrast of the STM image into account. This method was already applied successfully in previous works (Gura, L., *et al.* Continuous network structure of two-dimensional silica across a supporting metal step edge: An atomic scale study. *Physical Review Materials* **5**(7) (2021): L071001 and Lewandowski, A. L. *et al.* From Crystalline to Amorphous Germania Bilayer Films at the Atomic Scale: Preparation and Characterization. *Angewandte Chemie International Edition* **58** (2019) 10903-10908). Figure S2b shows the resulting neighborhood after a manual adjustment. The blue straight lines connect nearest ring neighbors. With the geometric information obtained from this network we calculate the idealized Si atom positions, which are plotted in Figure S2c. For visualization, Figure S2d shows the superposition of all these characteristic features. The calculated Si atom positions are located in the center of three adjacent ring centers.

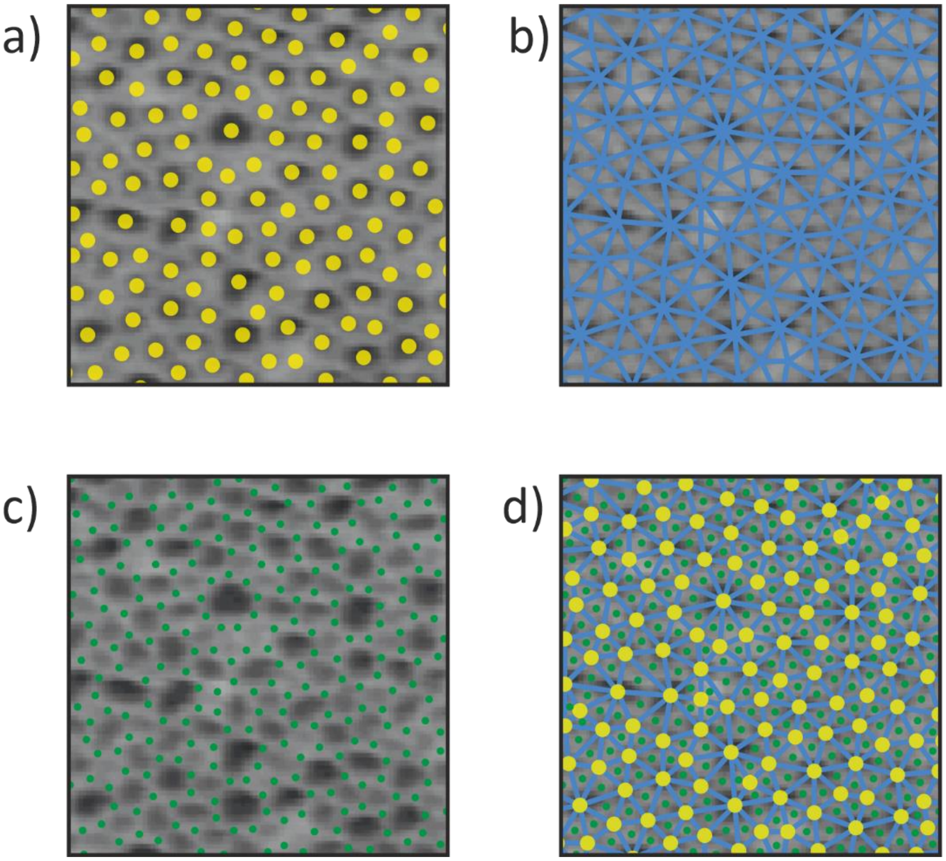

Figure S2: Semi-automated network detection on a 4 x 4 nm² cutout of Figure 3c. STM image with superimposed a) ring centers positions, b) ring neighbor connections, c) calculated Si atom positions, and d) all aforementioned features.

3. Si Atom Positions

Figure S3 shows the Si atom positions on the STM images from Figure 3 of the main manuscript. The positions are color coded with respect to their distance from the hole. The coordinate positions are given in Tables 1-3.

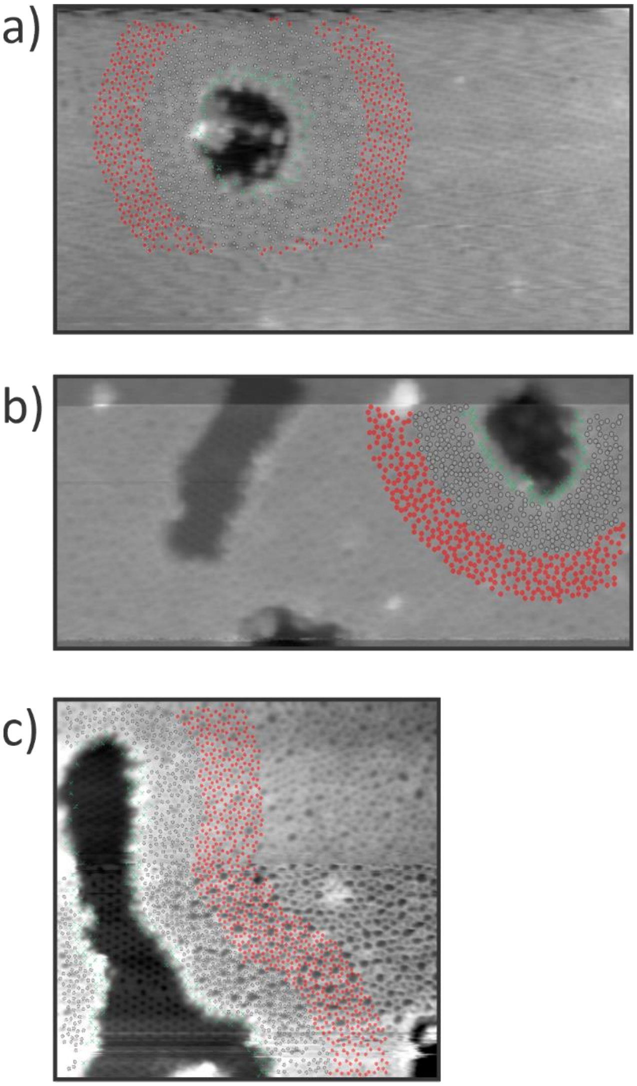

Figure S3: STM images of vitreous silica bilayer films with superimposed deduced Si atom positions. The color code for near (grey) and far (red) Si positions from the hole is similar to Figure S1. The STM images show the same scan as is Figure 3 in the main manuscript. a) STM image from Figure 3a, 30 x 17 nm². b) STM image from Figure 3b, 30 x 14.2 nm². c) STM image from Figure 3c, 20 x 20 nm².

Table S1: Si atom coordinates in nm for Figure S3a. The color code is similar to Figure S1 and S3, indicating atoms at the perimeter or buffer zone (green, _per), atoms in the far region (red, _far) and atoms in the near region (gray, _near).

x_per / nm	y_per / nm	x_far / nm	y_far / nm	x_near / nm	y_near / nm
7.30	10.47	5.37	14.13	12.73	12.53
7.37	10.77	6.27	5.83	7.60	8.63
7.50	10.20	6.87	5.27	13.43	10.00
7.60	11.47	11.17	4.30	10.70	13.53
7.67	11.77	4.83	12.90	11.43	13.30
7.70	9.67	16.03	9.73	13.30	9.23
7.73	12.07	6.80	15.60	8.13	7.70
7.77	9.33	10.83	4.20	7.27	11.93
7.77	11.10	4.27	10.43	13.47	10.53
7.87	10.07	4.30	10.97	6.97	10.97
7.97	8.80	7.13	15.77	12.90	12.33
7.97	12.63	4.73	8.83	7.17	9.90
8.07	8.57	6.10	15.13	7.87	13.23
8.10	12.20	4.87	8.40	8.27	7.43
8.17	12.90	4.90	13.20	6.87	10.33
8.40	8.03	12.83	4.73	7.40	9.07
8.43	8.47	11.17	16.17	9.40	6.83
8.53	13.07	15.67	8.00	13.30	11.80
8.57	7.77	12.13	15.93	7.40	12.40
8.80	7.57	15.97	9.20	7.77	8.20
8.90	12.93	14.03	5.23	7.53	12.87
9.27	13.23	5.47	14.40	7.10	11.47
9.30	7.67	15.50	7.13	11.80	7.17
9.67	7.20	15.87	8.60	12.13	13.27
9.70	13.10	8.27	4.47	12.03	7.23
10.00	13.30	15.43	6.87	8.73	7.03
10.23	7.17	15.63	7.67	10.33	13.77
10.27	13.23	7.37	4.83	13.17	8.30
10.47	7.47	14.57	14.63	10.77	6.80
10.60	13.00	12.53	4.63	11.00	13.67
10.80	13.13	6.57	5.40	13.57	10.27
10.97	7.30	16.07	11.67	12.80	12.83
11.07	13.07	5.00	7.90	13.00	7.57
11.13	7.63	4.40	9.37	12.60	13.07
11.37	12.73	4.60	12.30	12.53	7.20
11.50	7.57	5.83	6.27	7.17	12.27
11.57	12.90	13.57	15.47	7.50	8.37
11.60	7.97	6.50	15.53	9.80	13.87
11.83	12.87	5.67	6.50	13.63	11.00
11.97	7.73	4.73	8.53	11.67	13.50

12.13	12.70	11.47	16.20	13.47	11.53
12.17	12.20	14.93	14.27	8.67	13.67
12.27	7.73	4.20	9.93	9.47	13.83
12.27	8.73	15.87	12.60	7.03	11.77
12.33	12.50	6.97	5.00	10.23	6.53
12.37	11.87	16.00	12.13	7.33	8.83
12.43	9.13	16.13	9.43	13.70	10.73
12.53	7.90	4.83	8.13	6.67	10.53
12.63	8.37	5.13	7.37	6.73	10.83
12.67	11.23	4.13	10.67	13.17	12.37
12.70	11.93	12.00	4.30	8.13	13.57
12.73	9.80	16.03	8.87	7.33	12.73
12.77	10.50	15.67	13.13	9.53	6.57
12.87	11.63	4.43	12.00	6.83	10.00
12.90	9.37	5.00	13.80	7.10	9.37
13.03	10.07	16.23	11.13	7.57	13.20
13.03	11.03	4.10	10.20	13.57	9.47
13.07	10.70	14.83	14.53	8.90	13.80
10.00	13.60	15.03	5.87	6.90	11.27
11.17	13.37	4.97	7.60	13.63	9.73
12.33	12.97	14.50	14.90	12.30	7.03
11.13	7.00	16.30	10.67	11.47	6.80
10.37	6.83	10.70	3.97	7.87	7.57
13.37	11.17	13.97	15.37	8.23	7.13
10.47	13.53	16.27	10.20	8.47	6.93
7.33	9.63	15.43	13.67	7.90	13.57
8.50	13.43	13.40	4.70	7.70	7.83
9.23	13.60	15.33	6.27	13.40	12.13
11.90	13.23	4.20	11.47	12.83	7.23
		5.07	14.10	11.77	6.87
		7.47	4.60	13.43	8.83
		6.00	5.77	10.80	6.53
		16.27	11.40	9.93	6.43
		15.87	12.90	8.93	6.77
		14.70	5.47	13.43	8.53
		16.03	12.40	13.83	10.23
		16.17	11.93	13.83	11.20
		6.80	15.87	13.33	7.70
		16.00	8.33	8.47	13.90
		15.90	8.07	13.90	10.53
		4.47	8.83	13.07	12.93
		4.10	11.17	7.27	8.27
		5.27	6.87	6.53	11.00
		15.73	7.20	8.20	13.87

		15.80	7.47	11.17	13.93
		5.80	5.97	13.37	12.50
		16.33	9.93	13.77	9.23
		4.33	9.00	13.43	8.00
		15.60	6.63	10.53	6.33
		5.57	14.97	10.57	14.07
		4.50	12.83	13.77	11.57
		15.67	13.43	12.37	13.60
		5.93	15.37	13.90	9.80
		4.43	12.57	11.10	6.40
		13.07	4.50	11.40	6.50
		4.13	9.43	11.70	13.80
		11.67	4.03	6.90	12.43
		13.57	15.73	7.03	8.77
		14.27	5.03	6.53	9.93
		5.07	7.07	6.60	11.30
		4.03	9.70	12.67	13.47
		4.67	13.43	9.00	14.13
		5.27	14.63	9.87	14.23
		16.43	10.87	7.40	13.47
		14.97	5.57	6.73	11.97
		6.27	5.30	6.80	9.33
		16.43	10.43	12.27	6.77
		12.40	4.23	9.30	14.20
		15.27	14.20	14.00	10.03
		15.30	5.93	7.93	7.03
		4.47	8.53	7.03	12.90
		5.40	6.43	7.60	13.70
		15.57	6.33	9.13	6.37
		14.63	5.20	13.33	12.70
		16.27	8.70	6.30	10.43
		6.77	4.80	13.77	8.87
		15.47	13.97	13.63	12.30
		16.40	9.37	11.97	6.67
		6.27	15.77	13.00	7.00
		3.87	10.70	8.40	6.63
		5.67	15.27	7.70	7.20
		4.70	13.73	13.67	8.20
		4.20	12.13	6.70	12.27
		4.57	8.03	11.50	14.03
		13.57	4.53	14.10	11.13
		7.63	4.30	14.13	10.60
		16.23	8.40	7.20	7.93
		5.93	5.50	10.97	14.20

		8.03	4.13	13.93	11.80
		15.90	7.03	7.37	7.70
		15.03	14.70	8.83	6.47
		14.80	14.97	14.00	9.30
		16.03	7.57	10.10	14.40
		14.00	4.70	6.90	8.50
		16.10	7.87	10.40	14.33
		4.70	7.53	6.27	10.93
		5.17	6.60	9.90	6.10
		3.97	11.63	13.60	7.57
		16.10	13.00	6.97	13.13
		5.30	14.93	6.83	12.73
		3.80	10.13	7.13	13.40
		4.87	14.30	6.93	8.33
		7.13	4.47	14.07	9.60
		16.53	11.47	6.73	8.83
		15.87	6.73	12.03	14.00
		16.43	12.00	6.50	11.83
		6.00	15.70	9.13	6.17
		5.00	14.57	10.50	6.03
		16.30	12.50	6.43	11.53
		3.80	11.03	8.47	14.23
		15.27	14.50	6.17	10.17
		4.37	8.30	6.63	9.03
		5.53	5.90	7.90	6.80
		13.23	4.30	8.13	6.60
		4.00	11.97	12.33	13.93
		4.17	12.50	14.23	10.87
		4.30	13.07	13.33	7.03
		15.90	13.53	9.57	14.40
		3.93	9.23	13.23	13.27
		4.40	13.40	13.53	7.27
		4.83	7.00	6.53	9.43
		16.63	9.87	8.57	6.40
		16.47	8.83	8.07	14.17
		16.67	10.93	11.10	6.10
		4.03	8.93	11.53	6.23
		4.50	7.77	6.10	10.63
		5.33	6.10	8.80	14.37
		3.73	9.80	6.37	9.67
		16.53	9.10	14.27	10.10
		16.63	9.57	13.87	12.37
		14.23	15.67	7.53	13.97
		4.67	7.27	12.43	6.50

		6.83	4.50	11.97	6.40
		16.60	11.80	9.63	5.93
		16.33	12.80	10.20	5.90
		15.17	5.33	14.00	12.20
		16.50	12.27	9.37	5.97
		16.13	13.33	13.03	13.60
		6.17	4.97	14.33	10.37
		6.40	4.77	7.40	7.17
		16.40	8.20	7.03	13.57
		15.50	5.70	13.93	8.17
		15.77	14.00	8.27	14.37
		3.60	10.53	14.20	9.10
		15.77	6.10	7.23	7.40
		16.73	11.23	6.93	7.87
		16.77	10.47	12.97	6.60
		13.97	15.93	14.03	8.67
		16.17	7.10	14.33	11.37
		16.33	7.93	13.67	12.87
		14.87	5.00	14.23	11.67
		4.47	13.93	10.80	5.93
		3.57	10.27	11.37	6.00
		16.23	7.33	11.77	6.20
		7.30	4.13	6.40	12.37
		5.47	15.50	6.70	13.17
		3.60	11.10	6.57	12.80
		4.90	6.53	7.80	14.23
		5.63	5.40	12.73	6.43
		14.93	15.23	14.33	9.60
		15.97	13.83	6.60	8.57
		5.87	15.90	6.03	11.17
		5.47	5.60	11.57	14.37
		3.67	11.60	11.10	14.50
		5.10	15.13	6.37	9.17
		16.07	6.50	14.43	9.87
		16.87	10.77	6.13	11.47
		4.73	6.73	9.50	14.63
		15.43	5.43	6.73	8.07
		14.33	4.53	13.57	13.23
		3.97	12.70	14.23	8.83
		4.03	13.00	8.03	6.33
		3.67	9.27	8.87	14.63
		15.73	5.80	8.93	5.93
		15.47	14.70	8.50	6.13
		4.57	14.40	10.03	14.77

		3.57	9.60	12.23	6.27
		4.80	14.80	14.10	8.40
		4.20	13.57	7.63	6.63
		3.77	12.10	6.73	13.43
		15.13	5.07	14.53	10.87
		5.60	15.80	6.20	11.97
		14.67	15.63	9.20	14.73
		4.43	14.23	13.83	7.13
		4.07	8.17	6.33	12.63
		5.20	15.40	7.03	13.90
		3.83	8.70	10.63	14.70
		16.57	12.87	13.50	6.73
		16.93	10.00	13.97	12.77
		16.03	6.17	14.03	7.67
		3.50	11.40	6.50	13.03
		16.83	9.13	7.23	14.10
		16.73	8.57	12.10	14.37
		16.90	9.43	6.93	7.53
		4.23	13.83	14.43	9.33
		4.40	7.17	7.27	6.93
		16.87	11.87	9.17	5.73
		16.67	8.27	14.30	12.23
		15.83	14.30	9.73	14.83
		16.73	12.37	14.60	10.40
		5.03	6.00	11.40	14.60
		4.87	15.07	5.73	10.57
		3.57	11.83	10.10	5.60
		16.97	11.37	14.60	11.20
		14.80	4.67	11.33	5.77
		15.70	14.57	12.63	14.23
		16.53	7.73	14.43	11.90
		4.20	7.60	6.13	12.20
		17.00	10.27	5.73	10.13
		16.50	7.43	14.17	7.90
		16.43	13.40	7.40	6.67
		3.33	10.97	10.93	14.73
		16.40	6.87	13.33	6.50
		6.57	4.23	8.20	6.07
		4.80	6.23	5.93	9.60
		15.37	15.13	6.00	11.73
		4.53	14.73	10.77	5.63
		6.93	4.00	11.87	14.50
		3.87	13.20	7.73	6.33
		16.77	12.67	13.77	6.83

		3.27	10.70	14.70	10.67
		5.20	5.53	9.77	5.57
		4.50	6.67	8.67	5.90
		16.23	13.90	14.03	7.20
		3.63	12.33	7.70	14.50
		3.93	13.47	6.50	8.00
		17.03	11.67	8.20	14.70
		3.83	8.37	14.70	9.97
		6.27	4.40	6.30	8.53
		16.37	6.57	13.30	13.90
		3.70	12.63	5.73	11.13
		5.47	15.93	6.57	13.57
		16.93	12.17	10.23	14.97
		16.63	13.17	12.30	6.03
		3.47	9.07	10.50	14.93
		4.33	6.87	12.00	5.93
		17.17	10.83	6.07	9.07
		3.27	9.70	12.47	14.47
		3.97	7.77	14.20	7.47
		15.63	5.20	6.77	7.43
		4.13	7.30	12.83	6.10
		4.07	13.97	14.47	8.63
		3.20	10.03	6.80	13.97
		16.97	8.67	8.50	14.80
		15.33	4.87	11.07	5.57
		15.97	5.60	10.37	5.43
		5.47	5.03	5.60	10.83
		16.47	13.67	14.77	10.23
		4.17	14.27	13.80	13.47
		5.03	15.60	14.47	12.37
		17.13	9.50	14.40	8.33
		17.17	9.80	9.07	15.00
		5.67	4.80	14.23	12.83
		4.97	5.70	11.50	5.60
		17.03	8.93	5.63	9.83
		17.20	11.13	6.07	12.70
		3.50	8.77	7.20	14.40
		3.23	11.40	13.10	14.20
		16.87	8.07	6.27	8.23
		16.10	14.37	6.60	13.80
		5.20	15.87	6.23	13.13
		16.80	7.80	9.17	5.47
		17.27	10.37	5.50	10.33
		16.27	5.93	14.70	9.23

		3.13	11.17	7.90	14.77
		16.70	7.20	6.97	6.90
		5.13	5.30	14.83	11.30
		16.30	14.17	7.43	14.57
		4.67	15.30	12.57	5.93
		4.43	6.37	5.90	12.27
		3.30	12.00	9.50	5.37
		16.67	6.90	14.70	11.83
		4.37	14.90	6.00	8.77
		4.57	6.13	14.47	8.03
		14.83	16.00	6.27	13.40
		15.60	4.93	11.87	5.67
		3.03	10.60	14.47	12.60
		15.93	14.80	6.77	7.10
		15.90	5.27	11.07	15.00
		3.60	13.17	11.37	14.93
		3.13	9.47	13.93	6.63
		16.20	5.67	14.90	9.77
		17.37	10.63	7.57	6.13
		3.20	9.17	6.37	7.73
		3.00	10.20	13.70	13.83
		3.50	12.87	9.80	15.20
		3.33	12.30	13.53	6.27
		17.03	12.77	5.67	11.67
		3.73	13.70	6.03	12.97
		4.77	15.57	5.87	12.53
		16.57	6.30	5.53	11.37
		3.57	8.23	7.20	6.40
		17.20	12.27	15.00	10.63
		17.33	11.70	8.63	15.07
		16.90	13.27	12.00	14.80
		15.10	4.37	8.57	5.60
		17.33	9.30	5.77	9.27
		3.80	13.97	6.43	7.50
		15.67	15.33	12.40	14.73
		5.20	4.97	14.90	11.60
		3.70	7.70	14.73	8.67
		3.37	8.53	10.10	15.27
		4.43	15.23	14.73	12.27
		16.17	14.63	14.83	8.93
		4.70	5.67	14.30	7.00
		17.30	9.00	7.97	5.77
		4.00	14.53	14.13	13.37
		16.50	6.00	15.03	10.33

		17.43	11.23	14.93	9.43
		4.07	6.73	11.10	5.30
		17.20	8.43	6.67	14.23
		17.43	9.87	10.67	15.20
		17.13	8.13	14.33	13.10
		3.93	7.00	11.40	5.33
		15.37	4.50	13.10	5.90
		16.77	13.73	11.67	15.00
		17.00	7.60	6.93	6.57
		17.47	10.13	8.80	5.37
		17.07	13.07	7.00	14.57
		3.53	8.00	15.07	11.10
		4.10	14.83	7.27	6.20
		3.00	11.63	13.83	6.33
		15.20	15.93	6.37	13.90
		16.97	7.30	5.30	10.90
		17.23	12.57	14.87	12.10
		15.97	15.10	13.30	14.40
		4.50	5.83	14.23	6.73
		2.87	11.10	5.23	10.40
		2.83	10.80	6.03	8.07
		17.40	12.03	15.13	10.77
		4.20	6.30	14.53	7.37
		3.03	11.93	6.10	13.53
		17.50	11.50	10.43	5.10
		16.87	6.70	7.77	15.00
		16.57	14.23	13.40	5.97
		5.80	4.20	14.77	12.53
		16.97	13.53	10.93	15.23
		16.80	6.40	7.63	5.80
		15.60	15.63	7.47	14.90
		4.83	5.20	12.00	5.43
		4.03	6.50	6.10	7.73
		17.60	10.73	5.57	12.17
		3.40	13.40	8.13	5.53
		3.00	9.00	15.17	9.90
		4.63	15.77	5.30	9.70
		16.10	5.07	6.17	13.80
		2.77	10.00	6.73	14.50
		3.23	12.83	5.17	10.73
		3.47	13.70	6.40	14.13
		15.80	4.70	5.27	11.27
		16.77	14.03	14.53	7.07
		3.17	8.50	9.13	15.40

		17.63	11.03	9.53	5.03
		4.67	5.37	12.70	14.87
		2.83	9.43	6.47	7.00
		3.13	12.53	5.83	13.17
		16.43	5.47	5.60	12.60
		17.60	9.40	14.77	7.83
		17.43	8.50	5.67	8.67
		17.63	9.67	15.23	10.20
		5.43	4.37	5.43	11.90
		17.50	8.80	10.30	15.50
		3.60	14.20	12.57	5.53
		3.50	7.50	13.10	14.70
		4.23	15.40	14.03	13.93
		16.43	14.70	6.30	7.20
		3.70	14.50	10.90	5.03
		17.33	7.93	5.87	13.43
		16.70	5.80	5.47	9.17
		2.97	8.73	10.60	15.47
		2.70	11.30	9.53	15.50
		15.20	16.23	14.97	8.47
		3.30	7.93	11.47	5.10
		5.00	4.73	5.80	8.13
		17.27	7.67	13.03	5.60
		17.73	10.23	15.20	11.70
		16.63	14.50	14.30	13.63
		15.67	4.40	10.03	4.90
		3.67	6.90	14.90	12.67
		2.73	11.60	15.20	9.33
		2.67	9.63	15.13	8.87
		16.37	5.17	8.50	15.40
		17.33	13.13	11.73	5.13
		17.10	6.73	15.33	11.20
		17.17	7.03	7.17	5.93
		4.27	5.77	14.80	7.47
		2.77	9.17	5.00	10.13
		16.07	4.77	5.27	9.33
		4.10	6.00	15.17	12.00
		4.33	15.67	6.63	6.47
		17.63	12.13	13.67	14.47
		4.80	4.90	8.63	5.10
		2.57	10.63	15.33	9.63
		17.80	10.53	5.63	8.37
		3.90	15.07	14.67	13.23
		17.50	12.63	7.40	5.67

		16.67	5.53	14.43	6.50
		17.77	11.60	5.03	9.80
		2.53	10.27	14.07	6.10
		16.27	15.20	15.43	10.73
		15.80	15.80	6.90	14.90
		3.47	7.17	12.30	5.33
		3.10	8.17	5.37	12.37
		17.00	6.17	7.80	15.33
		3.27	7.67	15.00	8.07
		3.77	6.43	7.17	15.07
		3.10	13.33	12.77	5.40
		3.03	13.07	5.87	7.63
		16.47	14.97	5.07	11.43
		2.73	12.10	6.40	6.70
		3.97	15.33	9.23	4.83
		16.93	5.90	6.23	14.33
		4.40	5.30	5.53	13.07
		17.73	8.90	5.40	8.83
		17.90	11.10	15.40	11.53
		17.80	9.17	11.83	15.37
		17.63	8.30	15.20	8.60
		2.87	12.60	14.87	13.03
		3.23	13.90	14.73	6.87
		17.03	14.13	5.93	13.97
		17.33	13.57	15.47	10.30
		17.57	12.90	15.30	8.93
		17.70	12.43	11.13	4.87
		3.33	14.17	15.33	9.13
		17.83	11.90	5.40	12.80
		17.90	9.73	13.63	5.70
		17.60	8.00	11.13	15.57
		17.93	10.03	12.70	15.17
		5.03	4.37	6.03	7.17
		15.50	16.27	5.10	11.77
		4.20	5.50	6.50	14.73
		3.53	14.73	8.83	4.87
		17.93	11.40	4.83	10.77
		3.63	15.00	15.53	11.00
		4.27	15.93	15.20	12.50
		2.70	12.40	15.30	12.17
		16.87	14.60	5.67	7.83
		15.70	16.13	15.57	10.57
		3.53	6.57	8.10	15.53
		17.50	7.43	8.83	15.70

		17.43	7.13	14.60	13.57
		2.53	9.10	5.83	7.40
		2.83	12.90	14.37	6.17
		2.40	11.27	14.23	14.17
		3.70	6.20	6.60	6.17
		3.83	5.97	6.97	5.80
		17.30	6.50	14.70	6.57
		2.33	10.77	5.63	13.67
		17.23	6.27	5.97	14.23
		17.53	13.37	13.33	14.97
		2.40	9.57	13.97	14.50
		16.57	4.97	6.27	14.63
		2.47	11.70	7.97	5.13
		16.37	4.70	10.20	15.87
		16.30	15.53	15.03	7.27
		2.67	8.60	10.77	15.77
		4.57	4.77	14.00	5.77
		18.07	10.63	6.60	14.97
		16.10	15.80	14.97	6.97
		17.07	14.43	13.63	14.83
		16.77	14.90	15.60	9.60
		18.10	10.90	13.13	5.27
		2.83	8.13	6.73	5.90
		2.27	10.10	4.70	10.17
		16.87	5.30	11.93	4.87
		4.33	5.00	4.97	9.27
		2.90	13.57	7.30	5.40
		2.43	11.93	15.23	7.93
		3.00	7.60	11.57	15.67
		17.93	8.70	4.83	9.50
		2.97	13.83	5.63	13.90
		17.90	8.40	5.33	8.27
		18.10	9.53	4.77	11.33
		18.07	9.27	15.70	10.13
		2.23	11.03	5.17	8.73
		17.37	14.03	12.27	4.97
		2.33	9.30	15.53	8.90
		2.47	8.80	15.67	11.50
		2.23	9.77	6.13	6.60
		18.07	12.00	11.27	4.63
		17.17	5.70	15.57	9.07
		2.63	8.33	12.50	15.47
		17.80	7.83	14.20	14.43
		17.53	13.80	15.43	8.47

		3.13	14.40	5.97	6.87
		17.97	12.50	4.60	10.47
		17.83	13.00	10.17	4.47
		3.23	14.67	9.23	4.53
		3.70	15.53	9.57	15.97
		3.10	7.07	7.13	15.40
		16.83	5.03	12.90	5.10
		3.93	5.43	11.03	15.83
		2.23	11.53	5.03	12.43
		3.30	6.53	7.47	15.57
		3.77	5.67	4.63	11.00
		4.03	16.03	15.40	12.63
		18.20	11.50	8.20	4.90
		3.40	15.10	13.00	15.33
		17.63	6.90	12.27	15.57
		17.57	6.60	9.90	16.03
		18.23	10.10	5.43	7.77
		17.77	7.53	15.77	11.17
		4.80	4.17	5.27	13.27
		2.77	7.83	15.13	13.20
		17.10	5.37	15.77	9.83
		4.53	4.43	7.03	5.47
		2.07	10.63	4.87	11.90
		18.30	10.40	15.57	12.17
		17.43	6.03	15.83	10.57
		2.57	13.00	5.03	12.73
		3.43	6.10	15.70	9.27
		18.13	12.30	15.43	8.20
		2.40	12.50	14.87	6.37
		2.90	7.33	5.33	13.57
		17.00	14.97	11.80	4.63
		16.43	4.37	13.27	15.27
		2.03	10.33	5.57	7.30
		17.87	13.30	7.53	5.07
		16.57	15.67	9.10	16.03
		18.27	11.80	14.57	5.97
		4.10	4.93	13.67	5.30
		18.03	12.77	6.30	6.10
		18.17	8.77	5.10	8.43
		17.33	14.50	14.83	13.80
		18.23	9.03	4.93	8.93
		3.07	6.77	15.63	8.67
		2.80	14.03	15.30	7.33
		17.40	5.77	5.27	7.97

		2.20	12.03	6.07	14.80
		3.70	15.83	15.37	7.60
		3.90	5.17	15.80	11.70
		2.10	9.27	15.17	6.80
		18.10	8.17	15.40	12.97
		3.13	14.90	13.40	5.10
		3.23	6.30	15.73	12.00
		2.60	13.53	4.83	12.23
		18.03	7.90	9.40	4.33
		18.40	10.97	6.07	6.33
		3.37	15.37	14.73	14.10
		18.37	9.60	14.23	5.57
		1.97	11.07	4.57	9.53
		2.87	14.33	5.73	14.47
		18.40	9.90	15.63	12.47
		2.20	8.77	14.80	6.10
		1.97	9.73	15.93	10.30
		18.43	11.27	4.47	9.87
		2.20	12.33	8.67	4.47
		17.73	6.43	10.50	4.27
		17.57	14.30	5.37	7.47
		2.37	8.23	15.97	11.03
		3.50	5.63	6.50	5.70
		2.33	12.80	6.40	15.23
		17.67	6.13	9.80	4.27
		17.97	7.30	16.00	10.70
		17.90	7.00	5.70	6.83
		2.43	13.30	15.13	6.50
		1.87	10.80	4.60	11.80
		17.03	4.83	4.50	11.53
		17.37	14.77	13.93	15.07
		3.33	5.83	11.73	15.97
		2.03	9.00	14.50	5.67
		18.53	10.50	15.17	13.60
		1.93	11.60	5.53	7.00
		1.90	9.47	5.80	14.73
		2.50	7.77	14.20	14.87

Table S2: Si atom coordinates in nm for Figure S3b. The color code is similar to Figure S1 and S3.

x_per / nm	y_per / nm	x_far / nm	y_far / nm	x_near / nm	y_near / nm
21.60	28.05	25.50	20.70	1.91	1.55
21.68	27.60	18.83	26.63	1.41	2.00
21.75	28.28	24.15	20.85	1.81	1.56
21.90	27.30	20.48	23.55	1.54	1.77
21.90	27.90	22.28	21.75	1.67	1.63
22.13	26.48	28.35	21.60	2.13	1.62
22.28	26.18	22.58	21.60	1.69	1.62
22.28	27.23	26.10	20.63	1.96	1.55
22.43	26.78	19.13	25.73	1.43	1.93
22.50	25.80	26.63	20.70	2.00	1.55
22.58	26.10	23.25	21.15	1.74	1.59
22.80	25.65	24.38	20.70	1.83	1.55
23.18	25.28	19.95	24.00	1.50	1.80
23.55	24.53	19.28	25.20	1.45	1.89
23.55	24.90	23.78	20.85	1.78	1.56
23.85	24.45	20.93	22.80	1.57	1.71
24.30	24.75	18.45	28.05	1.38	2.10
24.60	24.68	25.80	20.55	1.94	1.54
24.68	24.08	27.23	20.78	2.04	1.56
24.75	24.30	21.30	22.28	1.60	1.67
24.90	23.78	28.20	21.30	2.12	1.60
25.28	23.70	28.88	21.90	2.17	1.64
25.58	23.93	19.58	24.45	1.47	1.83
25.88	23.70	18.90	25.95	1.42	1.95
26.18	23.78	21.75	21.83	1.63	1.64
26.18	24.08	18.38	27.68	1.38	2.08
26.40	24.38	24.90	20.48	1.87	1.54
26.48	24.08	19.05	25.43	1.43	1.91
26.78	24.68	20.70	22.95	1.55	1.72
26.85	24.38	26.40	20.48	1.98	1.54
27.00	25.13	29.18	22.05	2.19	1.65
27.00	27.00	27.83	20.93	2.09	1.57
27.00	27.38	25.20	20.40	1.89	1.53
27.08	24.75	27.60	20.78	2.07	1.56
27.15	26.48	19.20	24.90	1.44	1.87
27.15	28.05	21.98	21.60	1.65	1.62
27.23	25.73	27.00	20.55	2.03	1.54
27.30	26.85	19.65	24.08	1.47	1.81
27.38	25.35	20.18	23.40	1.51	1.76
27.38	27.68	28.73	21.60	2.15	1.62
27.45	26.33	23.03	21.00	1.73	1.58
27.53	26.03	19.95	23.63	1.50	1.77

		22.50	21.30	1.69	1.60
		21.08	22.20	1.58	1.67
		21.30	21.98	1.60	1.65
		18.45	26.48	1.38	1.99
		24.38	20.40	1.83	1.53
		29.55	22.28	2.22	1.67
		19.28	24.53	1.45	1.84
		29.40	22.05	2.21	1.65
		20.85	22.35	1.56	1.68
		22.73	21.08	1.70	1.58
		18.15	27.60	1.36	2.07
		21.45	21.75	1.61	1.63
		28.28	21.00	2.12	1.58
		23.78	20.48	1.78	1.54
		24.68	20.25	1.85	1.52
		28.13	20.85	2.11	1.56
		25.28	20.18	1.90	1.51
		25.80	20.18	1.94	1.51
		18.60	25.80	1.40	1.94
		23.10	20.70	1.73	1.55
		26.40	20.18	1.98	1.51
		19.50	23.85	1.46	1.79
		18.75	25.28	1.41	1.90
		21.98	21.30	1.65	1.60
		27.75	20.55	2.08	1.54
		20.40	22.80	1.53	1.71
		25.58	20.10	1.92	1.51
		20.18	23.03	1.51	1.73
		24.08	20.25	1.81	1.52
		18.83	24.98	1.41	1.87
		19.65	23.55	1.47	1.77
		22.20	21.15	1.67	1.59
		27.08	20.25	2.03	1.52
		21.00	21.90	1.58	1.64
		28.95	21.38	2.17	1.60
		23.48	20.40	1.76	1.53
		28.05	20.63	2.10	1.55
		26.10	20.03	1.96	1.50
		18.38	25.95	1.38	1.95
		18.00	27.23	1.35	2.04
		21.38	21.53	1.60	1.61
		28.65	21.00	2.15	1.58
		18.08	26.55	1.36	1.99
		18.53	25.43	1.39	1.91

		18.00	26.85	1.35	2.01
		18.98	24.38	1.42	1.83
		21.60	21.30	1.62	1.60
		22.50	20.85	1.69	1.56
		20.55	22.20	1.54	1.67
		24.68	19.95	1.85	1.50
		26.85	20.03	2.01	1.50
		29.55	21.68	2.22	1.63
		26.63	19.95	2.00	1.50
		25.20	19.88	1.89	1.49
		17.78	27.83	1.33	2.09
		22.20	20.93	1.67	1.57
		20.33	22.43	1.52	1.68
		18.68	24.75	1.40	1.86
		27.75	20.25	2.08	1.52
		19.20	23.78	1.44	1.78
		22.88	20.48	1.72	1.54
		19.58	23.25	1.47	1.74
		27.45	20.10	2.06	1.51
		25.50	19.80	1.91	1.49
		26.10	19.80	1.96	1.49
		24.08	19.95	1.81	1.50
		19.80	22.95	1.49	1.72
		22.65	20.55	1.70	1.54
		28.20	20.40	2.12	1.53
		21.08	21.45	1.58	1.61
		29.33	21.30	2.20	1.60
		23.40	20.10	1.76	1.51
		18.90	24.08	1.42	1.81
		20.85	21.60	1.56	1.62
		24.98	19.73	1.87	1.48
		28.73	20.70	2.15	1.55
		24.38	19.80	1.83	1.49
		18.30	25.28	1.37	1.90
		26.48	19.73	1.99	1.48
		21.45	21.08	1.61	1.58
		17.55	28.13	1.32	2.11
		23.03	20.18	1.73	1.51
		28.50	20.40	2.14	1.53
		20.40	21.90	1.53	1.64
		19.28	23.25	1.45	1.74
		19.05	23.55	1.43	1.77
		18.00	25.73	1.35	1.93
		28.05	20.10	2.10	1.51

		17.78	26.25	1.33	1.97
		18.38	24.75	1.38	1.86
		25.65	19.58	1.92	1.47
		17.55	27.23	1.32	2.04
		21.98	20.70	1.65	1.55
		20.03	22.35	1.50	1.68
		27.08	19.73	2.03	1.48
		27.38	19.80	2.05	1.49
		23.93	19.73	1.79	1.48
		18.23	24.98	1.37	1.87
		20.55	21.60	1.54	1.62
		25.95	19.50	1.95	1.46
		19.80	22.58	1.49	1.69
		17.40	27.68	1.31	2.08
		21.60	20.78	1.62	1.56
		21.00	21.15	1.58	1.59
		17.78	25.95	1.33	1.95
		22.43	20.33	1.68	1.52
		23.63	19.73	1.77	1.48
		18.00	25.43	1.35	1.91
		24.98	19.43	1.87	1.46
		17.48	26.70	1.31	2.00
		24.38	19.50	1.83	1.46
		18.53	24.08	1.39	1.81
		29.10	20.63	2.18	1.55
		17.33	27.45	1.30	2.06
		26.48	19.43	1.99	1.46
		17.40	26.93	1.31	2.02
		29.40	20.85	2.21	1.56
		18.23	24.53	1.37	1.84
		19.95	22.05	1.50	1.65
		19.13	23.03	1.43	1.73
		18.75	23.55	1.41	1.77
		20.10	21.83	1.51	1.64
		22.05	20.40	1.65	1.53
		28.05	19.80	2.10	1.49
		17.18	27.98	1.29	2.10
		25.20	19.28	1.89	1.45
		24.08	19.43	1.81	1.46
		22.80	19.88	1.71	1.49
		25.50	19.28	1.91	1.45
		26.18	19.28	1.96	1.45
		17.40	26.40	1.31	1.98
		18.53	23.78	1.39	1.78

		20.40	21.38	1.53	1.60
		27.15	19.43	2.04	1.46
		28.73	20.10	2.15	1.51
		24.60	19.28	1.85	1.45
		17.93	24.90	1.34	1.87
		23.33	19.58	1.75	1.47
		20.78	21.00	1.56	1.58
		18.23	24.23	1.37	1.82
		27.60	19.50	2.07	1.46
		21.38	20.55	1.60	1.54
		17.78	25.20	1.33	1.89
		18.68	23.33	1.40	1.75
		22.43	19.95	1.68	1.50
		18.83	23.10	1.41	1.73
		19.20	22.65	1.44	1.70
		19.43	22.43	1.46	1.68
		27.00	19.28	2.03	1.45
		17.48	25.80	1.31	1.94
		20.55	21.08	1.54	1.58
		26.70	19.20	2.00	1.44
		29.10	20.25	2.18	1.52
		22.88	19.65	1.72	1.47
		28.35	19.73	2.13	1.48
		27.90	19.50	2.09	1.46
		17.10	27.00	1.28	2.03
		17.03	27.30	1.28	2.05
		19.95	21.60	1.50	1.62
		19.65	21.98	1.47	1.65
		26.18	19.05	1.96	1.43
		17.85	24.60	1.34	1.85
		20.10	21.38	1.51	1.60
		27.45	19.28	2.06	1.45
		21.83	20.18	1.64	1.51
		21.60	20.25	1.62	1.52
		18.30	23.63	1.37	1.77
		28.73	19.80	2.15	1.49
		16.88	27.98	1.27	2.10
		20.78	20.70	1.56	1.55
		25.65	18.98	1.92	1.42
		17.10	26.33	1.28	1.97
		17.48	25.35	1.31	1.90
		23.33	19.28	1.75	1.45
		24.60	18.98	1.85	1.42
		18.00	24.00	1.35	1.80

		25.88	18.90	1.94	1.42
		25.13	18.90	1.88	1.42
		16.80	28.28	1.26	2.12
		18.38	23.33	1.38	1.75
		21.08	20.40	1.58	1.53
		23.93	19.05	1.79	1.43
		19.65	21.68	1.47	1.63
		19.35	22.13	1.45	1.66
		18.68	22.80	1.40	1.71
		17.10	25.95	1.28	1.95
		20.85	20.48	1.56	1.54
		22.20	19.73	1.67	1.48
		18.08	23.70	1.36	1.78
		18.90	22.50	1.42	1.69
		26.33	18.83	1.97	1.41
		22.65	19.43	1.70	1.46
		28.13	19.28	2.11	1.45
		27.23	18.98	2.04	1.42
		27.53	19.05	2.06	1.43
		21.90	19.88	1.64	1.49
		16.73	27.38	1.25	2.05
		16.80	26.85	1.26	2.01
		26.63	18.83	2.00	1.41
		17.63	24.45	1.32	1.83
		22.88	19.28	1.72	1.45
		20.25	20.85	1.52	1.56
		25.35	18.75	1.90	1.41
		16.65	27.68	1.25	2.08
		24.90	18.75	1.87	1.41
		28.43	19.35	2.13	1.45
		17.78	24.08	1.33	1.81
		19.95	21.08	1.50	1.58
		23.55	18.98	1.77	1.42
		20.40	20.63	1.53	1.55
		25.95	18.68	1.95	1.40
		21.45	19.95	1.61	1.50
		18.45	22.80	1.38	1.71
		24.30	18.75	1.82	1.41
		18.23	23.10	1.37	1.73
		16.73	26.55	1.25	1.99
		22.28	19.43	1.67	1.46
		21.15	20.03	1.59	1.50
		19.43	21.53	1.46	1.61
		24.00	18.75	1.80	1.41

		26.18	18.60	1.96	1.40
		16.58	27.08	1.24	2.03
		27.75	18.90	2.08	1.42
		17.18	25.13	1.29	1.88
		16.88	25.73	1.27	1.93
		28.05	18.98	2.10	1.42
		29.10	19.50	2.18	1.46
		17.33	24.53	1.30	1.84
		16.43	28.43	1.23	2.13
		19.73	21.00	1.48	1.58
		27.23	18.68	2.04	1.40
		26.93	18.60	2.02	1.40
		17.55	23.93	1.32	1.79
		20.33	20.40	1.52	1.53
		20.63	20.18	1.55	1.51
		17.78	23.48	1.33	1.76
		19.05	21.83	1.43	1.64
		21.75	19.58	1.63	1.47
		25.43	18.45	1.91	1.38
		25.73	18.45	1.93	1.38
		18.68	22.20	1.40	1.67
		16.35	27.75	1.23	2.08
		24.83	18.45	1.86	1.38
		22.73	18.98	1.70	1.42
		21.53	19.65	1.61	1.47
		19.43	21.23	1.46	1.59
		18.00	23.03	1.35	1.73
		16.88	25.35	1.27	1.90
		28.73	19.13	2.15	1.43
		17.10	24.75	1.28	1.86
		24.45	18.45	1.83	1.38
		16.28	28.13	1.22	2.11
		16.35	27.08	1.23	2.03
		18.23	22.58	1.37	1.69
		21.98	19.28	1.65	1.45
		27.60	18.60	2.07	1.40
		17.55	23.63	1.32	1.77
		21.00	19.80	1.58	1.49
		23.18	18.68	1.74	1.40
		17.25	24.15	1.29	1.81
		17.78	23.18	1.33	1.74
		19.05	21.53	1.43	1.61
		26.25	18.30	1.97	1.37
		29.10	19.20	2.18	1.44

		16.58	25.73	1.24	1.93
		20.70	19.88	1.55	1.49
		18.68	21.90	1.40	1.64
		16.35	26.33	1.23	1.97

Table S3: Si atom coordinates in nm for Figure S3c. The color code is similar to Figure S1 and S3.

x_per / nm	y_per / nm	x_far / nm	y_far / nm	x_near / nm	y_near / nm
0.55	12.45	7.35	12.70	7.30	5.30
0.55	12.80	8.95	7.55	1.65	18.00
0.60	10.00	8.55	8.45	4.60	9.55
0.60	13.40	11.00	5.90	11.05	1.75
0.75	13.70	7.00	18.20	11.60	0.55
0.80	7.95	13.25	3.70	4.65	9.00
0.80	9.30	12.00	5.35	5.60	7.85
0.80	15.05	9.80	6.25	5.90	7.50
0.80	15.55	8.80	8.05	10.75	2.90
0.85	10.80	7.50	15.95	10.85	2.70
0.85	12.05	7.95	9.20	4.85	16.75
0.90	12.35	7.55	16.20	0.45	10.60
0.95	8.35	14.30	0.45	4.55	13.25
0.95	10.00	8.45	8.70	9.50	3.65
1.05	9.60	6.35	19.05	1.95	5.30
1.05	14.35	6.70	18.65	1.00	1.05
1.10	7.45	12.85	4.55	9.00	3.65
1.10	11.90	13.65	2.80	1.30	6.20
1.10	17.65	7.65	13.40	1.00	6.55
1.15	6.75	13.50	3.05	4.50	10.60
1.15	7.90	7.15	11.05	0.75	8.20
1.15	10.60	7.40	17.60	4.90	8.75
1.15	16.55	12.55	4.95	10.95	2.10
1.20	7.20	9.30	7.30	1.00	7.00
1.20	10.25	9.60	6.90	1.45	2.70
1.25	8.15	7.70	15.00	0.65	7.75
1.25	8.85	13.50	3.30	5.10	13.75
1.30	9.50	12.40	5.15	5.45	8.05
1.35	7.70	7.65	13.10	11.65	1.15
1.35	9.20	13.15	4.20	1.70	3.95
1.35	17.40	7.20	11.30	11.25	1.55
1.40	11.40	8.80	8.35	0.95	0.80
1.45	1.00	7.55	9.80	1.85	5.85
1.45	6.75	7.60	15.45	4.65	10.35
1.50	6.30	10.40	6.30	5.05	8.40
1.50	7.10	7.40	17.85	6.20	7.35
1.55	6.60	13.95	2.55	7.40	5.50
1.55	8.25	7.85	14.05	7.60	4.90
1.60	2.00	7.70	16.80	8.30	4.20
1.60	17.60	7.85	14.55	1.90	4.40
1.65	0.65	7.30	10.80	1.90	4.70
1.65	9.25	7.40	12.30	0.80	17.50

1.80	6.10	9.90	6.50	1.35	2.05
1.80	7.65	9.55	7.15	1.35	2.25
1.80	8.75	7.65	17.25	6.55	6.95
1.85	3.10	7.40	10.10	0.75	7.50
1.85	3.40	11.60	5.85	1.45	3.80
1.85	7.15	10.65	6.30	4.80	13.30
1.90	1.15	12.80	4.90	5.85	7.80
1.90	2.15	7.55	12.50	7.25	5.75
1.90	2.45	14.40	1.65	10.60	3.30
1.90	6.70	12.15	5.55	0.55	9.30
1.90	8.10	7.70	15.75	1.05	6.30
1.90	9.05	9.85	6.75	4.65	17.20
1.95	3.80	11.20	6.10	4.80	15.90
1.95	17.40	7.15	18.45	6.65	6.35
1.95	18.00	6.95	18.70	4.65	12.75
2.00	7.35	14.20	2.25	4.75	9.80
2.05	3.55	7.95	14.35	6.90	6.10
2.05	5.60	13.15	4.50	1.25	3.40
2.05	6.35	7.65	17.50	0.45	9.65
2.10	2.80	7.80	15.25	1.25	3.15
2.10	4.85	9.20	7.75	4.60	11.55
2.10	5.10	8.60	8.90	5.25	14.30
2.10	8.60	13.90	2.85	6.70	6.65
2.15	17.70	13.50	3.85	10.20	3.55
2.20	8.30	14.55	1.15	1.00	8.70
2.25	2.05	8.20	9.35	8.60	3.90
2.30	1.70	7.95	14.80	5.00	16.15
2.35	5.60	7.35	11.85	10.00	3.70
2.40	5.30	9.15	8.00	2.00	18.30
2.45	3.55	10.25	6.50	4.85	15.15
2.45	3.85	7.85	16.30	0.80	8.90
2.45	4.40	12.45	5.40	3.70	18.15
2.45	4.70	11.45	6.05	1.40	18.20
2.45	17.65	13.45	4.10	4.60	12.45
2.50	6.35	7.95	13.45	7.95	4.55
2.60	5.95	7.85	12.95	1.15	18.05
2.65	18.25	9.40	7.60	2.30	18.40
2.70	4.15	7.85	17.05	10.85	3.15
2.70	5.25	7.90	16.60	4.70	11.05
2.70	17.90	14.65	0.60	4.95	15.60
2.75	4.90	13.75	3.40	5.25	14.00
3.05	17.70	7.45	11.40	3.05	18.45
3.40	17.60	8.05	13.90	4.65	11.85
3.50	17.15	8.50	9.20	11.10	2.70

3.65	12.10	7.50	12.05	11.90	0.90
3.65	16.00	9.05	8.45	4.80	9.30
3.65	17.75	11.85	5.95	11.90	0.55
3.70	10.35	7.60	10.25	1.70	5.30
3.70	10.65	7.55	10.75	4.10	17.65
3.70	12.50	12.10	5.80	11.70	1.45
3.75	11.45	14.45	2.05	9.15	3.95
3.75	14.80	10.85	6.45	9.45	3.95
3.80	11.20	6.65	19.30	11.15	2.35
3.80	15.30	7.90	9.85	0.50	8.25
3.80	15.70	7.60	18.05	1.30	5.90
3.80	16.90	11.10	6.35	1.55	5.80
3.90	9.05	14.65	1.45	4.80	10.80
3.90	13.15	9.75	7.30	5.10	14.85
3.95	9.40	14.25	2.60	5.30	8.50
3.95	14.55	7.80	12.50	11.45	1.70
3.95	15.10	13.75	3.70	1.05	1.65
3.95	16.25	7.50	11.65	5.25	13.50
4.00	9.90	8.10	13.65	0.70	6.55
4.00	10.40	13.00	5.05	1.15	2.65
4.00	11.60	7.40	18.50	7.85	5.00
4.00	11.95	7.65	10.55	8.55	4.30
4.00	12.65	8.05	13.15	8.70	4.10
4.05	10.80	8.20	9.65	5.15	8.90
4.05	11.05	8.90	8.90	7.35	6.00
4.05	13.60	10.10	6.85	0.70	7.05
4.05	15.55	14.80	0.95	1.10	2.40
4.10	8.55	7.95	12.70	1.65	4.25
4.10	12.95	10.30	6.70	3.50	18.40
4.15	10.15	9.30	8.20	0.60	17.70
4.15	16.90	8.00	15.80	0.70	0.70
4.20	9.60	7.10	18.95	8.05	4.75
4.20	10.65	8.05	16.10	10.45	3.70
4.25	8.90	8.05	15.35	0.35	7.75
4.25	8.90	9.65	7.60	4.75	17.45
4.25	15.30	7.90	17.65	4.95	10.30
4.25	17.25	8.25	14.30	4.95	13.10
4.30	12.45	7.65	11.20	7.20	6.20
4.30	15.60	7.60	18.35	10.65	3.60
4.35	8.15	9.10	8.70	1.65	4.90
4.35	11.20	13.40	4.70	6.90	6.80
4.35	11.45	14.10	3.10	5.55	8.35
4.35	12.10	14.00	3.35	4.85	11.30
4.35	13.65	7.90	10.10	6.80	7.10

4.35	14.55	13.00	5.25	5.25	16.90
4.40	16.20	8.10	17.10	0.60	7.25
4.45	13.00	7.85	17.95	1.00	3.05
4.50	7.95	11.60	6.30	1.20	3.95
4.50	8.45	13.30	4.90	1.50	5.55
4.50	10.10	8.30	14.05	7.70	5.60
4.50	15.05	8.25	14.90	1.00	3.50
4.50	16.50	14.90	0.45	4.95	12.80
4.55	15.85	12.70	5.60	1.05	1.95
4.60	13.90	10.80	6.70	5.30	16.15
4.65	14.25	7.90	12.25	0.80	6.15
4.80	16.40	12.30	5.95	6.45	7.60
4.85	7.90	13.70	4.30	5.20	13.25
4.85	8.55	7.80	12.05	6.10	8.00
4.90	7.60	7.75	11.00	2.95	18.75
4.90	13.65	8.20	16.65	5.05	9.85
5.00	14.40	10.55	6.80	8.95	4.15
5.15	8.10	11.85	6.25	0.55	6.80
5.30	7.55	8.75	9.35	5.10	9.20
5.50	7.35	8.25	15.20	11.50	2.00
5.55	7.05	14.70	2.15	0.25	8.05
5.75	6.10	10.05	7.30	1.45	18.50
5.75	6.40	7.05	19.25	5.35	8.80
6.00	6.85	7.40	18.85	0.35	9.00
6.10	6.60	7.75	11.55	0.65	18.00
6.15	6.05	11.30	6.55	4.15	18.15
6.30	5.65	12.90	5.50	5.50	13.65
6.30	7.05	8.15	15.60	1.75	18.55
6.35	6.35	10.20	7.10	2.30	18.75
6.70	5.55	8.25	16.85	0.95	2.80
6.75	5.05	8.40	14.60	4.30	17.90
6.90	5.30	13.65	4.60	9.60	4.15
6.95	5.80	9.50	8.30	5.10	10.15
7.00	4.60	14.50	2.80	5.55	13.90
7.05	4.90	8.35	13.25	4.95	12.00
7.25	4.20	11.55	6.50	11.85	1.65
7.30	4.45	9.00	9.20	0.50	8.75
7.35	5.05	14.05	3.80	11.40	2.30
7.45	3.95	7.85	11.35	0.60	6.30
7.60	4.60	8.15	17.60	1.10	5.75
7.75	3.95	7.95	10.55	6.85	7.30
8.00	3.40	8.50	9.75	10.10	4.05
8.00	3.75	8.25	17.30	0.35	8.50
8.00	4.25	9.85	7.75	0.95	3.75

8.30	3.90	12.60	5.90	5.55	14.60
8.40	3.15	15.00	1.65	11.35	2.90
8.70	3.30	8.25	12.65	11.15	3.30
8.75	3.65	8.45	13.55	9.95	4.15
9.10	3.05	8.35	16.15	1.45	4.35
9.20	3.50	8.00	18.15	5.30	15.60
9.30	3.25	14.45	3.05	5.50	16.45
9.65	3.20	8.75	9.60	2.55	18.90
9.75	3.55	8.10	10.25	5.05	10.65
9.85	2.90	13.95	4.25	4.95	12.30
10.00	2.05	7.90	18.40	8.00	5.25
10.05	1.65	12.30	6.20	0.85	5.85
10.15	2.95	8.40	16.45	5.40	14.95
10.35	2.75	15.15	1.10	6.75	7.55
10.40	1.50	9.40	8.80	5.80	8.45
10.40	2.20	12.10	6.35	1.20	4.20
10.45	1.10	14.25	3.55	8.65	4.55
10.45	2.55	8.00	10.95	1.40	4.75
10.55	0.30	13.30	5.35	0.80	3.30
10.65	1.70	15.00	1.95	6.40	7.90
10.65	2.05	11.00	6.90	12.25	1.10
10.70	0.95	11.25	6.80	0.85	2.30
10.75	0.55	10.10	7.60	6.05	8.30
10.90	1.15	7.95	11.65	7.15	6.80
10.90	1.55	8.45	13.00	7.95	5.50
11.00	0.50	9.55	8.60	11.35	3.15
11.15	1.00	8.20	12.25	10.40	4.00
11.25	0.75	8.60	13.95	4.75	17.80
11.35	1.15	8.00	11.85	5.05	17.50
		14.15	4.00	5.40	15.90
		10.45	7.10	8.35	4.85
		13.60	5.05	5.55	16.80
		9.75	8.20	11.70	2.00
		8.10	10.70	3.15	18.90
		8.40	15.65	7.65	6.10
		8.50	10.00	10.90	3.75
		8.50	15.35	0.80	2.10
		15.25	0.45	5.30	9.65
		14.90	2.45	7.80	5.95
		15.20	1.50	5.30	9.40
		7.65	19.00	4.55	17.95
		8.65	14.70	7.30	6.50
		9.90	8.00	5.20	12.65
		8.35	10.20	1.20	5.50

		13.20	5.60	3.75	18.70
		8.65	13.70	5.15	11.45
		8.35	12.40	5.45	15.45
		13.90	4.70	5.30	17.35
		12.70	6.10	11.15	3.60
		8.10	11.30	4.30	18.35
		13.55	5.25	8.95	4.45
		8.55	16.95	1.30	18.75
		9.30	9.25	5.60	8.90
		8.00	18.60	9.60	4.40
		14.85	2.75	11.65	2.60
		11.70	6.75	0.70	2.75
		15.35	0.95	0.75	18.45
		8.25	12.00	1.80	18.85
		8.55	17.25	5.45	13.05
		12.55	6.30	8.60	4.80
		8.30	18.10	12.10	1.75
		8.60	16.05	5.85	14.05
		8.40	17.85	8.30	5.15
		7.90	18.85	11.65	2.80
		9.50	9.05	2.05	18.95
		14.10	4.50	5.75	13.45
		8.65	16.50	9.95	4.40
		8.20	11.10	5.80	8.70
		8.80	14.15	5.85	14.60
		15.40	0.60	0.65	2.50
		8.60	15.80	5.25	11.85
		7.40	19.50	5.30	10.70
		8.20	11.45	10.85	4.00
		10.65	7.25	1.20	4.90
		11.50	6.95	5.05	17.80
		13.95	4.90	5.25	12.40
		8.85	14.45	7.15	7.40
		10.95	7.20	12.30	1.55
		12.15	6.65	3.50	18.90
		8.95	9.85	7.50	6.55
		8.75	15.25	7.70	6.35
		14.70	3.30	10.60	4.15
		13.30	5.80	1.10	5.25
		14.40	4.10	0.65	3.90
		10.40	7.70	11.85	2.20
		8.75	13.00	5.45	10.20
		9.85	8.65	5.55	15.20
		8.80	10.05	5.80	16.40

		8.75	16.80	0.55	3.25
		9.95	8.45	0.95	4.35
		8.85	15.00	2.55	19.20
		14.60	3.55	12.55	0.90
		15.30	2.10	0.70	5.60
		9.35	9.50	8.15	5.70
		14.30	4.40	7.30	7.15
		8.60	12.40	5.65	15.85
		7.70	19.35	8.05	5.95
		8.40	10.70	6.60	8.10
		11.95	6.85	3.00	19.20
		8.90	13.60	4.00	18.80
		8.80	16.25	6.95	7.80
		15.25	2.35	6.30	8.45
		8.30	18.55	1.10	18.85
		13.00	6.20	5.75	13.20
		10.20	8.10	4.25	18.65
		10.60	7.55	6.00	14.35
		8.45	18.30	5.45	12.80
		12.65	6.50	5.55	9.75
		15.50	1.65	9.45	4.60
		8.55	10.45	0.80	18.70
		9.20	9.80	4.60	18.25
		9.75	9.10	5.50	10.45
		8.80	12.65	5.70	9.15
		13.20	6.10	11.65	3.25
		10.40	7.95	5.60	9.30
		8.95	13.30	5.70	15.60
		13.90	5.35	0.70	4.20
		8.50	11.85	1.55	19.00
		8.75	17.70	0.45	2.95
		15.65	1.15	9.15	4.65
		8.50	10.95	5.85	16.05
		14.10	5.10	11.80	3.05
		11.55	7.20	7.20	7.65
		8.85	17.35	6.55	8.35
		8.10	19.10	11.40	3.75
		12.45	6.75	0.45	2.10
		9.95	8.90	11.90	2.50
		15.70	0.60	5.50	10.90
		9.10	14.05	6.90	8.00
		10.20	8.35	12.70	0.55
		14.65	4.05	5.50	11.35
		15.20	2.90	8.80	5.00

		8.50	11.55	5.60	17.50
		9.10	13.75	12.15	2.10
		11.10	7.45	0.40	5.65
		12.90	6.50	2.05	19.25
		14.50	4.50	8.50	5.35
		8.85	10.35	0.95	4.65
		8.70	12.05	2.80	19.40
		7.95	19.40	5.55	11.15
		15.00	3.35	5.65	10.00
		9.00	15.45	10.05	4.65
		15.60	1.95	5.90	17.10
		8.95	15.75	5.15	18.05
		9.15	14.65	3.50	19.20
		13.55	5.95	7.90	6.50
		9.65	9.55	10.65	4.40
		14.80	3.85	6.10	13.55
		9.15	14.95	11.05	4.20
		15.75	1.45	0.80	5.25
		9.05	16.25	6.05	8.90
		9.05	16.80	0.35	2.40
		8.70	18.30	5.55	12.40
		11.35	7.45	5.75	9.55
		15.20	3.15	6.15	13.85
		8.50	18.75	7.55	7.15
		12.00	7.15	2.30	19.40
		13.95	5.60	8.20	6.15
		15.85	0.95	6.05	16.65
		15.85	0.40	7.75	6.70
		15.50	2.55	9.05	4.90
		9.85	9.40	6.30	8.75
		10.90	7.65	6.05	14.95
		11.80	7.30	11.70	3.55
		13.45	6.20	5.90	15.20
		8.70	11.05	5.65	12.65
		13.80	5.85	8.45	5.65
		14.75	4.30	12.55	1.75
		9.30	14.20	3.25	19.40
		10.65	8.05	5.60	12.00
		9.20	13.20	4.00	19.10
		9.10	12.70	10.25	4.70
		15.45	2.80	1.35	19.20
		8.70	11.40	5.95	13.00
		14.35	5.15	4.80	18.45
		9.45	10.00	5.75	10.55

		9.15	15.95	6.00	9.15
		12.50	7.00	5.95	15.45
		10.20	8.95	6.10	16.05
		9.20	16.50	5.90	17.40
		9.35	14.50	7.40	7.75
		8.90	18.20	4.50	18.85
		9.70	9.80	11.45	4.00
		14.65	4.75	8.80	5.25
		9.05	10.50	9.60	4.90
		9.05	17.85	0.60	18.95
		10.45	8.50	10.50	4.65
		9.20	17.20	0.45	4.35
		8.35	19.30	12.90	1.15
		8.75	18.65	8.15	6.45
		12.30	7.20	11.30	4.20
		8.95	11.95	5.75	10.80
		10.90	7.90	10.95	4.45
		9.25	16.95	5.05	18.35
		13.10	6.70	6.15	17.00
		9.40	13.65	12.10	3.05
		9.30	12.95	0.70	5.00
		14.60	5.00	5.70	12.25
		10.40	8.80	12.35	2.25
		15.20	3.60	3.75	19.30
		11.50	7.65	5.45	18.05
		9.40	13.40	7.80	6.95
		15.10	3.85	8.45	6.10
		15.90	2.05	12.20	2.50
		10.65	8.35	7.05	8.25
		8.55	19.15	0.70	4.75
		13.55	6.40	5.90	10.05
		9.30	10.40	5.75	11.75
		11.80	7.55	6.40	14.40
		12.75	7.05	9.85	4.95
		9.45	15.10	0.25	5.40
		15.80	2.50	5.90	12.70
		9.50	10.25	4.75	18.75
		9.40	15.40	5.65	17.85
		10.15	9.45	6.85	8.50
		15.50	3.25	12.55	2.10
		9.40	16.55	8.60	5.85
		14.30	5.65	12.85	1.55
		9.55	14.05	6.30	16.55
		13.00	6.95	6.30	16.25

		9.15	12.05	13.05	0.50
		15.00	4.40	7.65	7.65
		16.15	0.40	7.75	7.40
		14.00	6.05	6.35	13.35
		10.30	9.25	11.95	3.65
		13.40	6.65	6.35	14.95
		14.45	5.50	6.55	8.90
		9.35	12.55	0.45	4.60
		16.15	1.45	6.05	9.65
		9.05	10.95	9.35	5.10
		9.30	12.30	0.75	19.25
		9.00	11.45	4.25	19.25
		9.60	13.80	5.90	11.20
		15.45	3.50	6.25	13.05
		15.75	2.90	12.30	2.75
		14.95	4.70	6.25	15.85
		9.15	10.75	6.00	10.45
		9.95	9.90	11.70	4.05
		16.00	2.30	7.45	8.05
		9.65	14.50	6.50	13.90
		9.00	18.70	0.40	5.10
		9.50	16.80	4.50	19.15
		12.35	7.45	6.20	15.55
		9.45	17.35	5.95	10.95
		9.15	18.40	6.05	10.25
		11.10	8.10	6.85	8.70
		11.50	7.90	7.30	8.25
		14.30	5.90	9.00	5.40
		9.10	11.60	6.10	9.85
		16.30	0.55	10.25	5.05
		15.30	4.05	6.20	9.40
		15.75	3.15	11.45	4.40
		9.40	17.85	12.25	3.30
		9.55	15.90	13.20	0.95
		9.75	10.30	12.15	3.55
		11.95	7.75	6.10	17.65
		9.75	14.25	10.65	4.90
		10.95	8.40	5.55	18.30
		10.20	9.75	10.00	5.15
		15.95	2.75	6.60	14.65
		15.25	4.30	11.10	4.70
		13.85	6.50	5.35	18.50
		9.65	13.00	11.95	3.95
		9.40	10.70	8.05	7.05

		12.80	7.35	6.65	14.15
		9.70	13.25	13.25	0.65
		16.25	1.95	5.95	17.90
		9.95	10.15	6.60	9.10
		10.70	8.95	8.45	6.60
		14.85	5.25	8.85	5.85
		9.65	16.40	8.65	6.35
		9.20	11.20	8.00	7.35
		9.75	15.00	13.05	1.75
		8.80	19.35	3.85	19.60
		14.05	6.35	6.10	12.50
		9.80	14.70	9.35	5.30
		9.55	17.65	6.05	11.75
		11.35	8.10	6.05	12.30
		10.60	9.25	12.80	2.25
		14.75	5.50	6.60	13.35
		13.20	7.15	6.10	11.40
		12.65	7.50	9.00	5.65
		12.25	7.70	6.40	12.85
		15.15	4.80	4.95	19.00
		9.70	16.85	11.70	4.35
		11.80	7.95	6.55	15.15
		9.05	19.05	7.85	7.85
		9.85	13.75	10.95	4.90
		9.70	15.60	6.60	16.70
		16.40	1.70	11.40	4.65
		13.60	6.90	6.50	17.20
		15.65	3.70	0.65	19.50
		9.40	18.40	10.50	5.15
		9.75	16.15	6.60	16.15
		11.00	8.60	4.75	19.25
		13.05	7.35	6.45	9.35
		10.90	8.85	6.45	15.40
		13.45	7.10	6.30	10.05
		9.75	17.30	6.55	15.85
		9.55	18.15	7.75	8.10
		15.95	3.30	6.20	10.90
		14.50	6.10	13.00	2.10
		15.60	3.95	4.20	19.60
		10.45	9.80	12.60	2.75
		13.85	6.80	6.75	13.65
		9.50	11.75	12.50	3.25
		9.85	16.60	8.40	6.90
		16.60	0.50	6.35	12.60

		9.80	12.60	5.80	18.35
		9.95	13.45	6.20	12.00
		15.45	4.50	6.30	10.65
		15.85	3.60	6.45	17.55
		9.85	10.60	9.95	5.40
		9.65	10.80	12.15	4.05
		9.85	17.05	7.05	8.95
		14.35	6.35	6.00	18.15
		16.25	2.75	9.00	6.05
		9.45	11.55	5.25	18.85
		14.90	5.65	13.45	1.15
		10.05	14.15	6.70	17.00
		8.90	19.65	12.65	3.05
		10.05	13.90	6.90	9.15
		9.30	19.00	7.55	8.50
		15.15	5.25	12.85	2.50
		10.75	9.45	6.30	11.40
		15.40	4.75	8.20	7.50
		10.00	15.10	6.80	16.55
		9.75	12.20	11.90	4.45
		10.65	9.65	8.95	6.35
		15.25	5.10	12.45	3.70
		16.45	2.35	13.55	0.60
		9.50	11.25	9.55	5.55
		16.70	0.95	6.80	13.15
		12.45	7.90	6.70	12.90
		12.75	7.75	6.95	14.65
		9.95	12.80	8.15	7.80
		9.50	18.70	6.40	11.15
		11.85	8.20	7.00	14.15
		10.10	14.65	6.70	15.65
		10.25	10.30	6.85	15.10
		11.55	8.35	6.85	16.25
		9.85	17.75	9.30	5.75
		14.75	6.05	11.05	5.15
		9.75	11.90	7.50	8.70
		16.20	3.25	7.85	8.35
		14.90	5.90	6.55	10.05
		10.45	10.15	9.75	5.60
		9.95	17.50	10.10	5.55
		16.60	2.20	10.60	5.40
		16.80	0.70	12.65	3.50
		11.25	8.75	7.30	8.95
		10.05	15.60	12.15	4.35

		16.35	3.00	6.65	9.55
		11.15	9.00	7.00	13.65
		10.20	14.85	13.65	1.00
		15.70	4.45	12.45	3.95
		14.45	6.55	7.00	14.85
		13.60	7.35	11.60	4.90
		10.15	10.60	6.55	10.60
		10.10	16.55	6.90	9.40
		15.20	5.55	13.70	0.75
		10.25	14.40	8.60	7.10
		13.20	7.65	6.60	12.45
		15.85	4.05	6.25	18.25
		10.20	13.45	5.95	18.60
		10.15	15.35	7.10	13.85
		15.50	5.00	8.45	7.40
		16.55	2.60	6.65	17.75
		10.00	12.35	13.30	2.25
		14.10	6.95	9.30	6.00
		9.85	18.25	6.95	17.00
		9.40	19.30	6.50	12.10
		11.45	8.65	7.15	14.40
		9.20	19.65	10.85	5.40
		10.80	9.85	6.70	9.80
		9.75	11.10	7.00	16.70
		10.15	16.05	11.90	4.75
		13.00	7.85	10.40	5.60
		16.05	3.85	9.10	6.55
		10.30	13.75	5.50	19.05
		16.85	1.70	6.70	10.30
		12.05	8.35	12.95	3.10
		12.45	8.15	9.90	5.75
		10.00	18.00	11.30	5.25
		13.45	7.60	6.85	12.65
		15.90	4.25	13.80	1.15
		10.20	17.00	7.15	13.45
		11.10	9.45	6.50	18.15
		14.35	6.85	13.15	2.65
		9.80	18.70	13.50	2.10
		10.70	10.15	7.10	13.20
		12.30	8.30	12.85	3.55
		10.25	16.30	6.65	11.15
		10.25	16.75	11.55	5.15
		10.25	12.80	8.10	8.40
		17.00	1.15	13.75	1.50

		15.15	5.95	8.35	8.00
		16.35	3.50	7.10	16.15
		11.25	9.25	5.85	18.90
		10.20	17.50	6.20	18.55
		13.85	7.40	6.65	11.55
		9.95	18.45	7.50	9.10
		17.05	0.70	12.35	4.50
		14.90	6.35	9.10	6.80
		15.90	4.60	7.70	8.90
		10.40	13.25	8.95	7.00
		16.30	3.75	13.00	3.35
		14.10	7.25	7.10	15.35
		10.30	15.80	7.05	15.65
		17.05	1.50	8.30	8.25
		16.65	3.00	6.95	17.65
		10.50	14.35	12.70	4.10
		14.80	6.55	6.80	10.75
		16.95	2.10	5.35	19.50
		10.05	11.75	13.15	2.90
		11.10	9.75	7.10	9.60
		15.45	5.65	9.35	6.50
		15.75	5.10	13.40	2.55
		13.00	8.10	7.15	17.20
		10.50	14.95	7.30	14.95
		10.35	17.25	14.00	0.75
		16.80	2.75	7.40	14.35
		12.75	8.25	12.10	4.90
		10.00	11.20	7.20	15.90
		10.55	13.85	9.50	6.25
		10.45	13.00	7.05	12.60
		10.30	12.30	8.70	7.60
		10.45	15.45	7.40	13.80
		15.40	5.85	12.30	4.75
		12.00	8.65	11.30	5.50
		15.15	6.25	6.85	11.00
		10.25	18.00	6.80	12.05
		16.10	4.40	7.30	16.35
		9.75	19.30	7.30	16.65
		10.45	10.80	8.60	7.90
		9.90	19.00	7.00	9.85
		10.85	10.30	14.05	1.00
		17.20	0.50	7.15	17.45
		15.95	4.85	9.95	6.00
		11.75	8.80	10.45	5.90

		13.65	7.80	13.00	3.75
		10.35	17.75	6.85	11.40
		10.10	11.50	11.05	5.65
		10.65	14.10	12.65	4.40
		10.65	14.50	8.15	8.70
		17.25	1.00	9.35	6.75
		14.60	7.00	7.00	10.35
		13.90	7.65	14.10	0.55
		10.45	12.50	13.95	1.70
		11.50	9.30	8.00	8.90
		10.50	15.75	7.60	9.30
		10.35	12.05	7.45	13.55
		16.65	3.50	6.75	18.30
		10.60	15.30	11.75	5.35
		14.30	7.35	12.95	4.00
		10.55	16.35	7.35	13.00
		10.55	16.65	7.35	15.30
		10.70	14.80	12.10	5.10
		10.35	11.10	6.90	11.80
		12.45	8.60	7.55	14.05
		14.85	6.85	7.50	14.85
		17.15	2.25	10.25	6.05
		11.70	9.05	7.55	14.55
		16.80	3.30	13.25	3.40
		15.75	5.60	9.05	7.30
		10.70	13.40	13.80	2.25
		16.50	3.95	7.45	9.55
		13.20	8.25	6.05	19.15
		15.90	5.35	6.45	18.75
		10.75	13.65	7.40	17.10
		10.30	18.50	7.45	16.80
		17.05	2.75	7.10	12.30
		11.30	9.95	7.10	17.95
		11.15	10.20	14.20	1.15
		10.60	17.25	7.10	10.60
		14.55	7.25	10.70	6.00
		12.70	8.55	13.95	2.05
		10.95	10.55	14.15	1.50
		10.70	12.90	11.55	5.60
		16.35	4.40	7.15	10.15
